# Genetic variation of macronutrient tolerance in *Drosophila melanogaster*

**DOI:** 10.1038/s41467-022-29183-x

**Published:** 2022-03-28

**Authors:** E. Havula, S. Ghazanfar, N. Lamichane, D. Francis, K. Hasygar, Y. Liu, L. A. Alton, J. Johnstone, E. J. Needham, T. Pulpitel, T. Clark, H. N. Niranjan, V. Shang, V. Tong, N. Jiwnani, G. Audia, A. N. Alves, L. Sylow, C. Mirth, G. G. Neely, J. Yang, V. Hietakangas, S. J. Simpson, A. M. Senior

**Affiliations:** 1 Charles Perkins Centre, The University of Sydney, Camperdown, NSW 2006 Australia; 2 School of Life and Environmental Sciences, The University of Sydney, Camperdown, NSW 2006 Australia; 3 Cancer Research UK Cambridge Institute, University of Cambridge, Cambridge, UK; 4 Molecular and Integrative Biosciences Research Programme, Faculty of Biological and Environmental Sciences, University of Helsinki, Helsinki, Finland; 5 Institute of Biotechnology, University of Helsinki, Helsinki, Finland; 6 School of Biological Sciences, Monash University, Melbourne, VIC 3800 Australia; 7 Section of Molecular Physiology, Department of Nutrition, Exercise and Sports, Faculty of Science, University of Copenhagen, Copenhagen, Denmark; 8 Department of Biomedical Sciences, Faculty of Medical and Health Sciences, University of Copenhagen, 2200 Copenhagen, Denmark; 9 School of Mathematics and Statistics, The University of Sydney, Camperdown, NSW 2006 Australia; 10 Present Address: Stem Cells and Metabolism Research Program, Faculty of Medicine, University of Helsinki, Helsinki, Finland

**Keywords:** Cell signalling, Functional genomics, Metabolism

## Abstract

Carbohydrates, proteins and lipids are essential nutrients to all animals; however, closely related species, populations, and individuals can display dramatic variation in diet. Here we explore the variation in macronutrient tolerance in *Drosophila melanogaster* using the *Drosophila* genetic reference panel, a collection of ~200 strains derived from a single natural population. Our study demonstrates that *D. melanogaster*, often considered a “dietary generalist”, displays marked genetic variation in survival on different diets, notably on high-sugar diet. Our genetic analysis and functional validation identify several regulators of macronutrient tolerance, including *CG10960/GLUT8*, *Pkn* and *Eip75B*. We also demonstrate a role for the JNK pathway in sugar tolerance and de novo lipogenesis. Finally, we report a role for *tailles*s, a conserved orphan nuclear hormone receptor, in regulating sugar metabolism via insulin-like peptide secretion and sugar-responsive *CCHamide-2* expression. Our study provides support for the use of nutrigenomics in the development of personalized nutrition.

## Abstract (web summary)

Responses to diet composition may be linked to susceptibility to metabolic diseases such as type 2 diabetes. Here the authors report that *Drosophila melanogaster* displays genetic variation in survival on different diets and describe the importance for JNK-pathway and a conserved orphan nuclear hormone receptor *tailless* in regulating sugar tolerance.

## Introduction

Carbohydrate, protein, and lipid are the major energy yielding components of food. After ingestion, macronutrients are metabolized by pathways that allocate nutrients to cellular maintenance and growth. Intracellular nutrient sensors detect different dietary inputs and orchestrate an integrated adaptive response ensuring energy homeostasis^1,2^. An ancient, and well-known sensor of amino acids is mTOR. The ChREBP/Mondo-Mlx transcription factor complex has been shown to serve as an intracellular sugar sensor that activates responses such as glycolysis, lipogenesis, and circadian rhythm^3–5^. Lipid sensors include the Peroxisome proliferator-activated receptors (PPARs) and Liver X receptor (LXR)^6,7^. Metabolic pathways are highly conserved across evolution. For example, glycolysis, the first step in the breakdown of glucose, is conserved in virtually all eukaryotic and prokaryotic cell types.

While metabolic pathways are conserved, there is considerable variation between human populations and ethnic groups in the prevalence of metabolic diseases, such as type 2 diabetes (T2D). For example, the Pima people of Arizona (USA) have perhaps the highest prevalence of T2D of any population^8^. Among Greenlanders, a variant of *TBC1D4/AS160*, a mediator of insulin-stimulated glucose uptake, confers a 10-fold increased risk of T2D^9^. Similarly, a variant of *CREBRF* is associated with body mass index (BMI) in Samoans, a population where obesity is prevalent^10^. Furthermore, a variant of a monocarboxylate transporter *SLC16A11* explains ~20% of the increased risk of T2D among Mexican and Latin American populations^11^. Arguably, such genetic variation arises because of adaption to nutritional environments. For example, whereas the traditional diet of Pima people was high in carbohydrates, Inuit people were primarily exposed to high-fat foods^12,13^. These differences are today reflected in the genes associated with metabolic diseases in these populations. For example, *TBC1D4/AS160* plays an important role in insulin-stimulated glucose uptake, which has not been essential in the Greenlandic environment where most foods were high in fat. Interactions between such genes and modern Western diets may help explain the increasing prevalence of metabolic disorders^14,15^.

Genetic reference populations have been developed to identify gene-by-environment interactions in controlled experiments^16^. One such panel now exists in the fruit fly *Drosophila melanogaster*, which has emerged as an important model in the study of nutrition and metabolic disease. Like humans, flies develop obesity and insulin resistance in response to high-sugar and high-fat diets^17,18^. Importantly, the molecular mechanisms of several metabolic diseases appear to be conserved in the fly^19^. We used the *Drosophila* Genetic Reference Panel (DGRP) to examine the genetic basis for variation of macronutrient tolerance. The DGRP consists of ~200 fully sequenced *D. melanogaster* (hereafter *Drosophila*) strains derived from a single outbred population^20^. By subjecting the strains to six diets differing in their macronutrient composition, we found that there is substantial inter-strain variation in survival on different diets. Variation in survival was maximized on high-sugar and high-coconut oil diets, whereas most strains thrived on high-protein, high-lard, and high-starch diets.

We identified a number of candidate genes mediating survival under different nutritional conditions. In vivo validation revealed several genetic regulators of macronutrient tolerance and metabolic homeostasis, which were not previously described in such roles. For example, we demonstrate a role for *Protein kinase N* and *Eip75B* in organismal sugar tolerance. We show that loss of *CG10960*, a putative *GLUT8* homologue, leads to a dependence on dietary sugar for growth. We describe a role for the JNK pathway in regulating sugar tolerance and sugar-induced de novo lipogenesis. Finally, we show that *tailless*, a highly conserved orphan nuclear hormone receptor, is required for survival on a high-sugar diet. We demonstrate that *tailless* regulates the expression of a nutrient-responsive hormone *CCHamide-2* in the fat body, and loss of *tailless* in the fat body leads to suppression of *Drosophila* insulin-like peptide (dILP) secretion and reduced growth.

## Results

### Genetic background determines survival on different diets

**Fig. 1:**
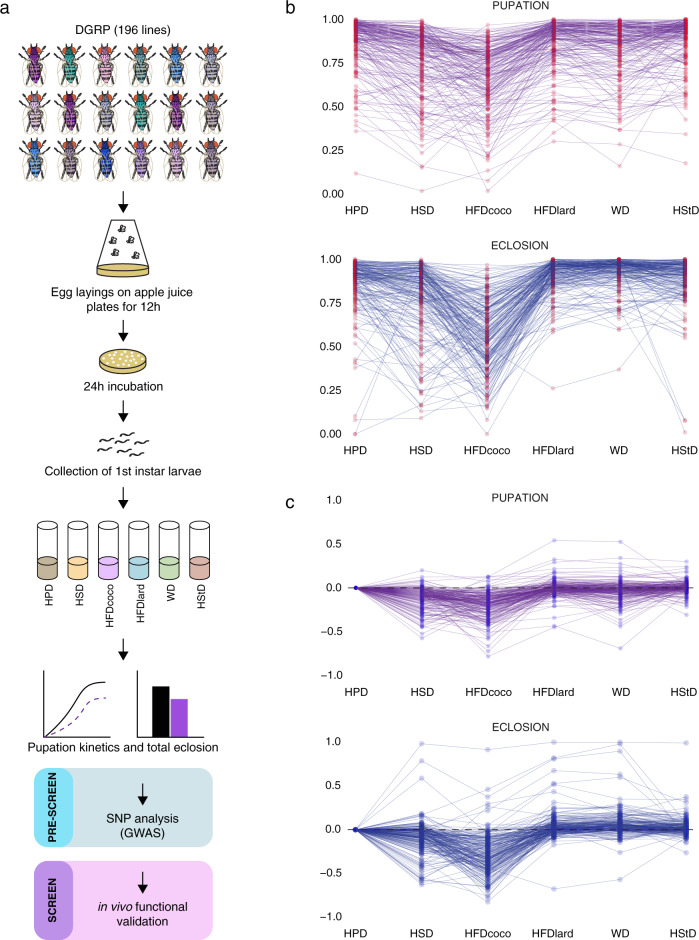
Experimental outline and survival of DGRP across the six diets. **a** The experimental outline. 196 lines of the *Drosophila melanogaster* genetic reference panel (DGRP) were raised on six different diets: high-protein (HPD), high-sugar (HSD), high-fat-coconut-oil (HFDcoco), high-fat-lard (HFDlard), Western (WD), and high-starch (HStD). First-instar larvae were collected onto the diets in a controlled density of 30 larvae per vial, and at four replicate vials per diet. The experiment was run in cohorts of 10 strains (all diets at the same time). Pupation was monitored daily to establish pupation kinetics of each strain across the diets and finally total eclosion was scored two weeks after. HPD was composed of 10% (w/v) dry baker’s yeast, and the other diets had the same amount of yeast as a base. HSD was supplemented with 20% (w/v) sucrose, HFDcoco with 20% (w/v) coconut oil, HFDlard with 20% (w/v) lard, WD with 10% (w/v) sucrose and 10% (w/v) lard, and HStD with 20% (w/v) potato starch. **b** Survival of the 196 DGRP strains to pupal stage (pupation) and to adult (eclosion) across the six diets. Pupation rate standard deviations (among lines) of 0.194 (HSD), 0.188 (HFDcoco), 0.162 (WD), 0.145 (HPD), 0.144 (HFDlard), and 0.140 (HStD). Eclosion rate standard deviations (among lines) of 0.211 (HSD), 0.203 (HFDcoco), 0.166 (HPD), 0.138 (HStD), 0.099 (HFDlard), and 0.092 (WD). **c** HPD-normalized survival of the 196 DGRP strains to pupal stage (pupation) and to adult (eclosion) across the other five diets. Source data are provided as a Source Data file.

To determine if gene-diet interactions affect macronutrient tolerance in *Drosophil*a we studied the survival of 196 DGRP strains across six diets: high-protein (HPD), high-sugar (HSD), high-fat-coconut-oil (HFDcoco), high-fat-lard (HFDlard), Western (WD), and high-starch (HStD) (Fig. 1a). All six diets had the same protein base (baker’s yeast), supplemented with either sucrose, starch, coconut oil, lard, or a combination of sucrose and lard (“Western diet”). The energy content of the diets was measured with a bomb calorimeter (Supplementary Table 1). High-fat diets had the highest energy content (HFDlard = 9.4 Mj/Kg, HFDcoco = 9.25 Mj/Kg) whereas high-starch and protein-only diets had the lowest energy content (HPD = 2.25 Mj/Kg, HStD = 3.98 Mj/Kg).

**Fig. 2:**
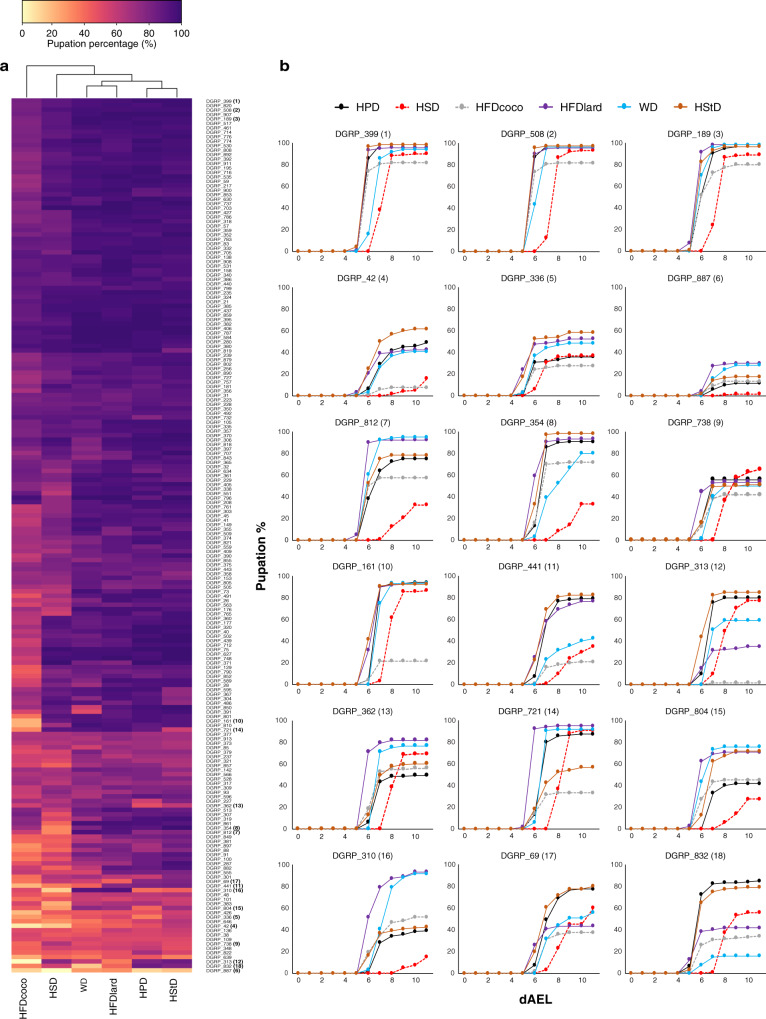
Diet-dependent survival of DGRP strains into pupae. **a** A heatmap showing the survival of the 196 DGRP strains into pupal stage across six different diets. **b** Pupation kinetics of selected DGRP strains. The numbers after DGRP strain IDs correspond to the ones indicated in the heatmap, *n* = 4 vials (each with 30 larvae) per diet and genotype. dAEL = days after egg laying. Source data are provided as a Source Data file.

Development from larvae to pupae (pupation) and from pupae to adult (eclosion) showed variation between the 196 strains tested, with most variation observed for HSD and HFDcoco diets (Figs. 1b, c, 2a and Supplementary Fig. S1a, b). The HFDlard resulted in the most rapid pupation time, with the slowest being HSD. Thus, the fat composition in lard seems to be beneficial to flies during development (Supplementary Fig. S2a). Equally good survival was observed for HPD and HStD across the strains (Supplementary Fig. S2b). The two diets that yielded the poorest survival were HSD and HFDcoco diets, with 76 and 67% of animals surviving to pupation, respectively (Supplementary Fig. S2b). However, a number of strains showed poor survival on HPD and HStD, but relatively good survival on HFDlard, HFDcoco, and HSD diets. Exemplar pupation curves for strains displaying diet-dependent survival are shown in Fig. 2b. We estimated the amount of among-strain variance in the response of survival through pupation to diet (treating HPD as the reference diet) via generalized linear mixed models (GLMM; Supplementary Tables 2 and 3). GLMM1, which assumed homogeneous responses to diet among the strains (i.e., no genotype-environment interaction or ‘GxE’) had poorer fit than GLMM2 which assumed heterogeneous responses (i.e., GxE; ΔAIC_GLMM2-GLMM1_ = −3411; *χ*^2^ for Δ deviance = 3451, DF = 20, *p* < 0.001; Supplementary Table 4). In summary, distinct isogenic *Drosophila* strains derived from the same founder population vary in their ability to survive on different diets suggesting that genetic components determine tolerance to different macronutrients.

### GWAS to pre-screen candidate genes affecting nutrient tolerance

**Fig. 3:**
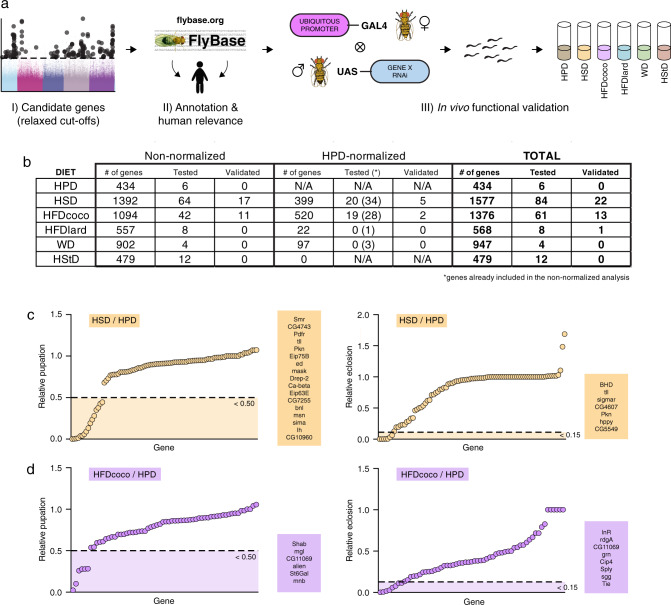
In vivo functional validation screen. **a** A schematic outline of the functional validation screen. Please see FlyBase for further information: https://flybase.org/^86^. **b** A table presenting the number of genes identified with one or multiple SNPs associated with altered survival, the number of genes tested in functional validation screen, and the number of genes validated in the screen. **c** Plots for relative pupation and eclosion (HSD/HPD) of candidate genes. The dashed lines (0.5 for pupation and 0.15 for eclosion) present the chosen cut-off values. **d** Plots for relative pupation and eclosion (HFDcoco/HPD) of candidate genes. The dashed lines (0.5 for pupation and 0.15 for eclosion) present the chosen cut-off values. *n* = 2–3 vials (each with 30 larvae) per diet and genotype.

To screen for genetic factors driving diversity in macronutrient tolerance, we performed a multivariate GWA analysis to identify candidate SNPs and genes. We performed the GWAS on both non-normalized and HPD-normalized data. To achieve the most comprehensive analysis of diet responsive genes, and because we were performing an in-depth functional validation of all selected candidate genes, we performed the GWA analysis with relaxed cut-offs (unadjusted MANOVA *p*-value < 10^−5^, Wilcoxon Rank Sum Test *p*-value < 0.01, and absolute difference in median phenotypes of at least 0.3 (unnormalised) or 0.2 (normalized); Supplementary Fig. S3). Three criteria were used in selecting genes for the functional validation: (1) statistical significance (as described above); (2) previous biological annotation (FlyBase and literature); and (3) a known human orthologue (Fig. 3a). For the functional in vivo analysis, we chose to use the GWAS data based on pupation (rather than eclosion), which yielded the highest number of candidate genes with SNPs (for lists of all SNPs, see the Source Data file). Moreover, in terms of nutrient utilization, the transition from larval to pupal stage is the most critical; pupation is dependent on the so called “critical weight”, which is directly determined by ability to store nutrients^21^.

The number of genes with SNPs associated with altered survival per diet is presented in Fig. 3b. Based on the non-normalized data we selected 139 genes for functional in vivo validation and based on HPD-normalized data a further 39 (Fig. 3b; see Supplementary Data 1 for the aggregated results of the functional validation).

### Functional in vivo validation screen of candidate genes

To validate candidate genes, we performed an in vivo functional screen involving whole-body knockdown of each gene and testing their survival on the diet(s) identified in the GWAS (Fig. 3a, b and Supplementary Data File). For example, genes that were associated with poor survival on HSD were knocked down and the survival was assessed on HSD and compared to survival on HPD (the base diet). We used the *Tub-GAL4* driver to obtain strong ubiquitous knockdown. In a case of early developmental lethality, a *Ubi-GAL4* driver that is also ubiquitous but weaker in strength than *Tub-GAL4*, was used as an alternative. Of the 165 genes tested (eight were candidates for more than one diet) 36 were validated. We used a cut-off of <0.5 for pupation phenotype hits (i.e., <50% of total pupation relative to pupation on HPD). Although candidate genes were selected based on pupation, some genes displayed an eclosion phenotype upon knockdown. To further interrogate potential genetic regulators of macronutrient tolerance, we also focused on genes with a strong eclosion phenotype (cut-off of <0.15) (Fig. 3c, d and Supplementary Data 1).

### Coconut oil is detrimental for the development of *Drosophila*

Our functional in vivo screen validated 13 genes for HFDcoco. These genes were *alien*, *CG11069*, *Cip4*, *grn*, *InR*, *mgl*, *mnb*, *rdgA*, *sgg*, *Shab*, *Sply*, *SiaT* (*ST6Gal),* and *Tie* (Fig. 3b, d). The validated genes were tested across all 6 diets to further confirm the diet-specificity of the phenotypes (Supplementary Fig. S4). When retested across all 6 diets, some of the HFDcoco hits did not display as strong a phenotype as in the original in vivo validation screen. Although still significant, the effect on pupation on HFDcoco was almost null in the case of *mgl*, *CG11069* and *alien* (Supplementary Fig. S4), and phenotypes of *SiaT* (*ST6Gal)* and *rdgA* did not repeat in the second round.

We observed decreased survival in most of the control animals (kk, GD, and Trip RNAi library background strains crossed with *Tub*- or *Ubi-GAL4*-driver) on HFDcoco (Supplementary Fig. S4). Moreover, the high variation observed in the survival of DGRP strains on HFDcoco (Figs. 1b, c, 2a and Supplementary Figs. S1a, b, S2b) suggests that coconut oil may have adverse effects on the development and survival of flies in general. In contrast, the diet supplemented with lard (HFDlard) appeared to be beneficial for development, resulting in relatively fast pupation and good overall survival (Supplementary Figs. S2a and S4). In line with this, the number of genes associated with poor survival was low on HFDlard compared to HFDcoco. We tested eight of these genes, and found that the knockdown of only one gene, *Lactate dehydrogenase* (*ImpL3*) led to reduced survival on HFDlard (Supplementary Data 1). To conclude, we identified several genes associated with poor survival on HFDcoco. However, the adverse effects of coconut oil on overall survival of wildtype DGRP strains and RNAi control flies make dissecting true gene-diet interactions on this type of high-fat diet challenging.

### Identification of genetic regulators of nutrient tolerance

For HSD, we chose 84 candidate genes to be further tested in vivo. Out of the 84 genes tested, 22 were validated, including *BHD*, *bnl*, *Ca-beta*, *CG10960/GLUT8*, *CG4607*, *CG4743*, *CG5549*, *CG7255*, *Drep-2*, *ed*, *Eip63E*, *Eip75B*, *hppy*, *Ih*, *mask*, *msn*, *Pdfr*, *Pkn*, *sigmar*, *sima*, *Smr,* and *tll* (Fig. 3b, c and Supplementary Data File). When further tested across all six diets, all hits, except for *Ih*, repeated (Supplementary Fig. S4). The confirmed hits are all for genes with no previous links to sugar tolerance, and thus we decided to focus on characterizing these HSD hits in detail.

**Fig. 4:**
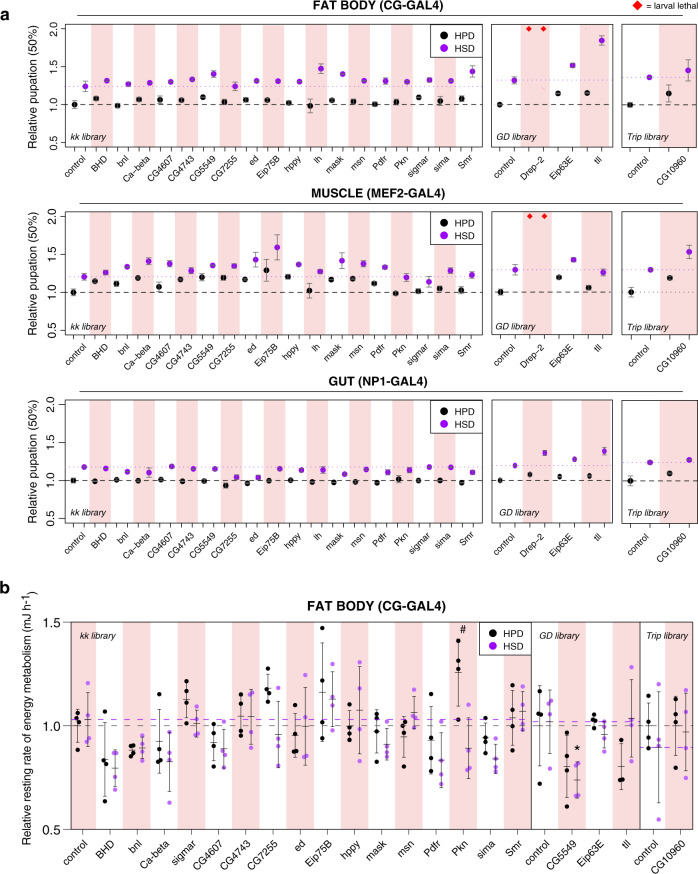
Tissue-specific metabolic phenotypes of the HSD hits. **a** The relative point (normalized to control) at which 50% of larvae pupated under tissue-specific knockdowns (*Cg-GAL4*, *Mef2-GAL4* and *NP1-GAL4*) of HSD hits. *n* = minimum of 3 vials (each with 30 larvae) per diet and genotype. Error bars display SD. See Source Data file for statistical analyses and exact *p*-values. **b** Relative resting rate of energy metabolism (mJ h^−1^) of *Cg-GAL4* knockdown of HSD hits (normalized to control HPD), *n* = minimum of 3 flies per diet and genotype. Error bars display SD. Statistical significances were calculated using the two-way ANOVA in conjunction with Dunnett’s multiple comparisons test (by genotype) and with Šídák’s multiple comparisons test (by diet). Data are presented as mean values +/− SD. * *p* = 0.0348 (Dunnett’s multiple comparisons test), # *p* = 0.004 (Šídák’s multiple comparisons test). Source data are provided as a Source Data File.

We next explored the tissue specificity of these genes in the context of sugar sensitivity. First, we examined the role of each gene on development using three different tissue-specific drivers: the fat body (*Cg-GAL4*), muscle (*Mef2-GAL4*), and midgut (*NP1-GAL4*) (Fig. 4a). These three tissues have key roles in regulating metabolism in flies^22^. Knockdown of *Drep-*2 in the fat body or muscle led to lethality in larvae on both HPD and HSD diets (Fig. 4a). Tissue-specific knockdowns of our genes of interest resulted in developmental delays under many conditions, however, the most robust effect was seen with the fat body-specific knockdown of *tailless*, which resulted in a severely delayed development on HSD (Fig. 4a). This suggests a key role for *tailless* in regulating sugar tolerance during development in the fat body.

The metabolism of *Drosophila* larvae and adults differs greatly. The larval stage is characterized by a ~200-fold increase in body mass accompanied by highly anabolic metabolism, including high expression of glycolytic enzymes and TCA cycle components^23^. During the transition from pupal to adult stage the levels of HNF4 transcription factor and its targets, including mitochondrial OXPHOS genes increase^24^. Therefore, we hypothesized that the genes identified to be critical in larval sugar tolerance might show distinct phenotypes in adults. We first established the role of our genes of interest in regulating starvation sensitivity after consuming either HPD or HSD. The ability to tolerate energy shortage likely reflects nutrient storage efficiency and nutrient resource allocation during starvation. We used the same three tissue-specific drivers, *Cg-GAL4*, *Mef2-GAL4* and *NP1-GAL4*, for knockdown. Notably, both fat body- and muscle-specific knockdown of *mask* led to decreased survival under starvation (Supplementary Fig. S5a), although the phenotype was strongest in muscle specific HPD-fed knockdown flies. The fat body-specific knockdown of *tailless* (*tll*) led to a starvation sensitivity on both HPD and HSD diets (Supplementary Fig. S5a), suggesting that fat body *tailless* also plays a key role in the metabolism of adult flies.

Next, we examined whether these genes play a role in regulating whole-organismal rates of energy metabolism (metabolic rate) in adult flies. Genetic manipulation of important metabolic regulators can affect metabolic rate. For example, overexpression of the *Drosophila* insulin-like peptide dILP1 can increase metabolic rate in *D. melanogaster*^25^. We knocked down our genes of interest in the fat body and collected one-day-old male flies and fed them HPD or HSD for 2 days. We then estimated the resting metabolic rate (MR, mJ h^−1^) of individual flies. The resulting MR data were normalized to account for body mass and activity (Supplementary Fig. S5b, c). Dietary sugar content had a negligible effect on MR in control flies (Fig. 4b). Only the knockdown of *CG5549* resulted in significantly reduced MR on HSD when compared to control, however, a diet-dependent reduction in MR was observed upon knockdown of *Pkn* (Fig. 4b). A diet-dependent reduction in body weight was observed upon knockdown of *BHD*, *Pdfr,* and *sima* (Supplementary Fig. S5b). Moreover, knockdown of *tll* and *CG10960/GLUT8* resulted in significantly lower body weight on both diets (Supplementary Fig. S5b). We also looked at activity levels and found that knockdown of *BHD*, *bnl*, *msn,* and *Pdfr* significantly reduced the overall activity of adult male flies (Supplementary Fig. S5c).

### *CG10960/GLUT8* is required for survival on sucrose-free diets

**Fig. 5:**
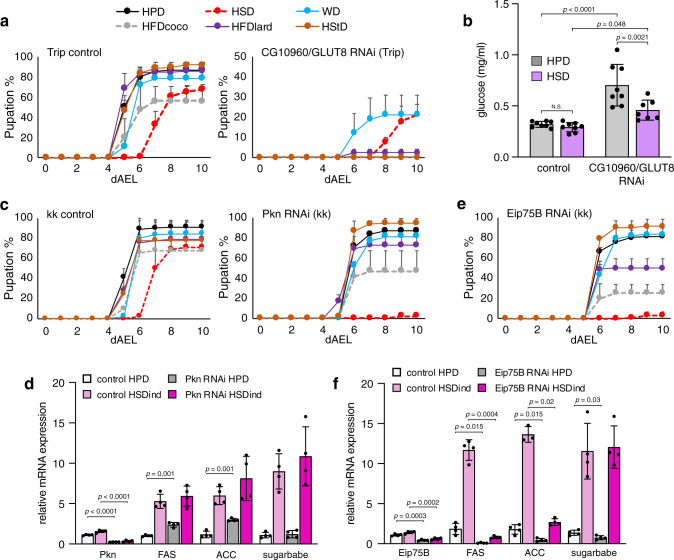
CG10960/GLUT8, Pkn, and Eip75B regulate sugar tolerance in *Drosophila*. **a** Pupation kinetics of *CG10960/GLUT8* RNAi and Trip control (*Tub-GAL4* > ) animals on six different diets, *n* = minimum of 3 vials (each with 30 larvae) per diet and genotype. **b** Circulating glucose levels of *CG10960/GLUT8* RNAi and Trip control (*Ubi-GAL4* > ) larvae on HPD and HSD (10% sucrose), *n* = minimum of 7 (each with 15 larvae) per diet and genotype. **c** Pupation kinetics of *Pkn* RNAi and kk control (*Tub-GAL4* > ) animals on six different diets, *n* = 3 vials (each with 30 larvae) per diet and genotype. **d** Whole-body expression of *Pkn*, *FAS*, *ACC,* and *sugarbabe* in control and *Pkn* RNAi (*Tub-GAL4*>) larvae after transient (8 h) HSD feeding (HSDind), *n* = 4 (five second-instar larvae per sample) per diet and genotype. **e** Pupation kinetics of *Eip75B* RNAi (*Tub-GAL4*>) animals on six different diets, *n* = 3 vials (each with 30 larvae) per diet and genotype. **f** Whole-body expression of *Eip75B*, *FAS*, *ACC* and *sugarbabe* in control and *Eip75B* RNAi (*Tub-GAL4*>) larvae after transient (8 h) HSD feeding (HSDind), *n* = 4 (five second-instar larvae per sample) per diet and genotype. Statistical significances were calculated using the two-way ANOVA in conjunction with Tukey’s multiple comparisons test (**b**) or unpaired two-tailed Student’s *t* test assuming unequal variances (**d** and **f**). Data are presented as mean values +/− SD. Source data are provided as a Source Data File.

One of the most interesting phenotypes was observed with the whole-body knockdown of *CG10960*. The closest mammalian homologue for *CG10960* is *GLUT8* (*SLC2A8*). We found that ubiquitous loss of *CG10960/GLUT8* resulted in early lethality on sucrose-free diets (Fig. 5a and Supplementary Fig. S4). Interestingly, the *CG10960/GLUT8* knockdown larvae were hyperglycaemic both on HPD and HSD (10% sucrose) (Fig. 5b), suggesting an increased tolerance of elevated circulating glucose levels. The mammalian *GLUT8* is a ubiquitously expressed dual-specificity glucose/fructose transporter that has been implicated in the mediation of the effects of high-fructose-induced glucose intolerance and dyslipidemia in mice^26^. Intriguingly, *GLUT8* knockout mice are resistant to fructose-induced glucose intolerance^26^, suggesting a conserved role for the fly *CG10960*/*GLUT8*.

### *Protein kinase N* is required for survival on HSD

Our in vivo screen identified a gene called *Protein kinase N* with extreme sugar intolerance. *Pkn* RNAi larvae were largely unable to pupate on HSD, while maintaining pupation on other diets (Fig. 5c). We have previously shown that sugar intolerant Mondo-Mlx deficient flies are unable to induce the expression of de novo lipogenic (DNL) genes *FAS* and *ACC*^3,27^. Interestingly, the loss of *Pkn* did not affect the induction of *FAS* and *ACC* upon transient HSD feeding (Fig. 5d), suggesting that induction of DNL genes is not the only mechanism underlying sugar tolerance. Furthermore, the induction of Mondo-Mlx target *sugarbabe* was normal in *Pkn* RNAi animals (Fig. 5d), suggesting that Pkn regulates sugar tolerance in *Drosophila* independent of Mondo-Mlx.

### *Eip75B* regulates both in vivo sugar tolerance and DNL

*Eip75B*, a gene encoding a nuclear receptor, was found in our screen to be required for sugar tolerance (Fig. 5e). Interestingly, the survival of *Eip75B* RNAi animals was reduced also on both high-fat diets relative to controls (Fig. 5e). We looked at the expression *FAS* and *ACC* upon transient HSD feeding and found that *Eip75B* RNAi animals were unable to induce DNL genes in response to HSD (Fig. 5f). Intriguingly, the expression of *sugarbabe* was normal in *Eip75B* RNAi animals (Fig. 5f), suggesting that there might be yet another mechanism independent of Mondo-Mlx regulating sugar tolerance and DNL gene expression in flies.

### JNK signalling moderates sugar tolerance and induction of DNL genes

**Fig. 6:**
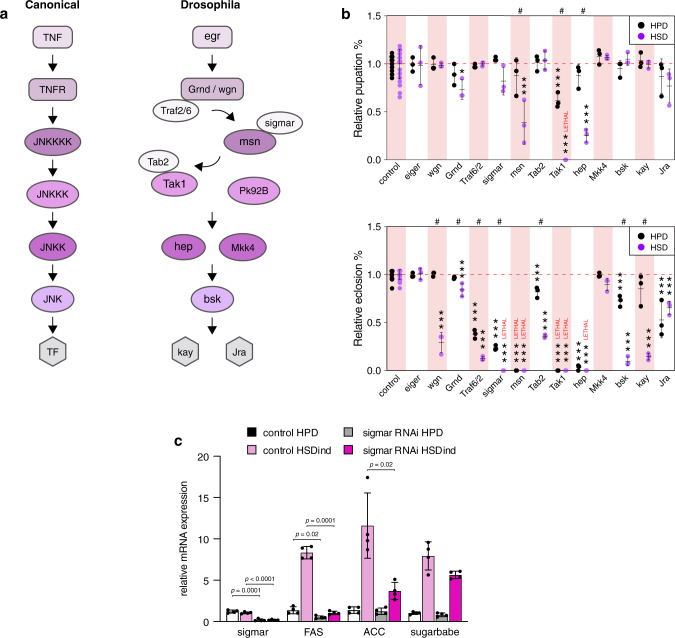
JNK signalling is required for dietary sugar tolerance and DNL. **a** JNK pathway is well-conserved in *Drosophila*. **b** Knockdown of *wengen* (*wgn*), *grindelwald* (*Grnd*), *TNF-receptor-associated factor 6* (*Traf2/6*), *sigmar*, *misshapen*, *TAK1-associated binding protein 2* (*Tab2*), *TGF-β activated kinase 1* (*Tak1*), *hemipterous* (*hep*), *basket* (*bsk*) and *kayak* (*kay*), all lead to a significantly reduced survival on HSD. # = significantly lower survival on HSD vs. HPD (within genotype). *n* = minimum of 3 vials (each with 30 larvae) per diet and genotype. Data for control animals (*Tub-GAL4*> kk control 60100, *Tub-GAL4*> GD control 6000, *Tub-GAL4*> Trip control 36303, *Ubi-GAL4*> kk control 60100 and *Ubi-GAL4*> Trip control 36303), is pooled in the figure. Statistical significances for each knockdown were however calculated against their respective library control line. Statistical significances were calculated using the two-way ANOVA in conjunction with Dunnett’s multiple comparisons test (by genotype) and with Šídák’s multiple comparisons test (by diet). * *p* < 0.05, ** *p* < 0.01, *** *p* < 0.001 (Dunnett’s multiple comparisons test), ^#^ *p* < 0.05 (Šídák’s multiple comparisons test). See Source Data file for exact *p*-values. **c** Whole-body expression of *FAS* and *ACC* in control and *sigmar* RNAi (*Tub-GAL4*>) larvae after transient (8 h) HSD feeding (HSDind), *n* = 4 (five second-instar larvae per sample) per diet and genotype. Statistical significances were calculated using an unpaired Student’s t test assuming unequal variances. Data are presented as mean values +/− SD. Source data are provided as a Source Data File.

The c-Jun N-terminal kinase (JNK) pathway is an evolutionarily conserved stress signalling pathway (Fig. 6a), activated by multiple stimuli ranging from DNA damage and reactive oxygen species to inflammatory cytokines^28,29^. The outcomes of JNK activation depend on the specific context and can vary from cell death to cell proliferation and survival. Among our hits were two known regulators of the JNK pathway, *misshapen* and *sigmar*^30^. Loss of both *misshapen* and *sigmar* led to a similar sugar-sensitive phenotype (Supplementary Fig. S4). As *misshapen* and *sigmar* are known modulators of the JNK pathway, we next asked if the JNK pathway is required for sugar tolerance. We knocked down every known step of the pathway, and found that in addition to *misshapen* and *sigmar*, loss of *wengen* (*wgn*), *grindelwald* (*Grnd*), *TNF-receptor-associated factor 6* (*Traf2/6*), *TAK1-associated binding protein 2* (*Tab2*), *TGF-β activated kinase 1* (*Tak1*), *hemipterous* (*hep*), *basket* (*bsk*) and *kayak* (*kay*) all led to a reduced survival under HSD when compared to HPD (Fig. 6b). Interestingly, we did not observe reduced survival upon knockdown of *eiger* (*egr*), the *Drosophila* TNF ligand (Fig. 6b). We also looked at the expression of DNL genes *FAS* and *ACC* in *sigmar*-knockdown larvae after transient sugar feeding. Whereas sugar feeding induced a high expression of *FAS* and *ACC* in control larvae, this induction was nearly abolished in *sigmar*-knockdown animals (Fig. 6c). We also examined the expression of *sugarbabe* and found it to be normally induced on HSD upon knockdown of *sigmar* (Fig. 6c), suggesting that the JNK pathway controls the expression of DNL genes independent of the Mondo/Mlx-sugarbabe axis. To conclude, our results show that the JNK pathway is required for dietary sugar tolerance and sugar-induced expression of the DNL genes in *Drosophila*.

### *Tailless* is required in the fat body for dietary sugar tolerance

**Fig. 7:**
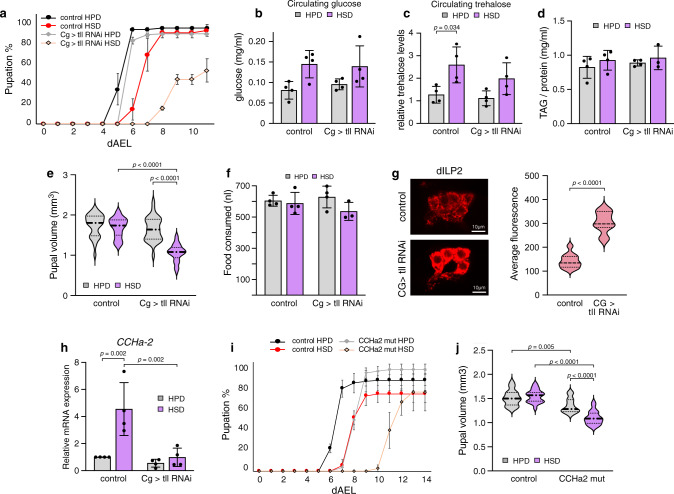
*Tailless*, a nuclear orphan hormone receptor, regulates dietary sugar tolerance in the fat body. **a** Pupation kinetics of control and *tailless* (*tll*) knockdown (*Cg-GAL4*>) animals on HPD and HSD, *n* = 3 vials (each with 30 larvae) per diet and genotype. Circulating glucose (**b**) and relative trehalose (**c**) levels in haemolymph of control and *tll* knockdown (*Cg-GAL4*>) pre-wandering third-instar larvae raised on HPD or HSD. *n* = 4 (each with 10 larvae) per diet and genotype. **d** Triglyceride levels of control and *tll* knockdown (*Cg-GAL4*>) pre-wandering third-instar larvae raised on HPD or HSD. *n* = minimum of 3 (each with 10 larvae) per diet and genotype. **e** Pupal volumes of control and *tll* knockdown (*Cg-GAL4*>) raised on HPD and HSD, *n* = 30 per diet and genotype. **f** Food consumed by control and *tll* knockdown (*Cg-GAL4*>) pre-wandering third-instar larvae on HPD and HSD, *n* = minimum of 3 (each with 10 larvae) per diet and genotype. **g** dILP2 accumulation measured by immunostaining of control and *tll* knockdown (*Cg-GAL4*>) pre-wandering third-instar larvae raised on a standard laboratory diet, *n* = 11 per genotype. **h** mRNA levels of *CCHa2* in fat bodies of control and *tll* knockdown (*Cg-GAL4* >) pre-wandering third-instar larvae after transient (8 h) HSD feeding as measured by quantitative RT-PCR. *CDK7* was used as a reference gene, *n* = 4 (3 fat bodies per sample) per diet and genotype. **i** Pupation kinetics of control and *CCHa2* mutant larvae on HPD and HSD, *n* = minimum of 3 vials (each with 30 larvae) per diet and genotype. **j** Pupal volumes of control and *CCHa2* mutants raised on HPD and HSD, *n* = 20 per diet and genotype. Statistical significances were calculated using the two-way ANOVA in conjunction with Tukey’s multiple comparisons test (**b**–**f**, **h**, and **j**) or two-tailed Mann–Whitney *U*-test (**g**). *p*-values < 0.05 were used to denote a significant result. Data are presented as mean values +/− SD (**a**–**d**, **f**, **h**–**i**) or as mean values +/− SE (**e**, **g**, **j**). Source data are provided as a Source Data File.

Next, we focused on the role of *tailless* in dietary sugar tolerance. *Tailless* is a well-conserved orphan nuclear hormone receptor with a role in neuronal stem cell regulation^31–33^. In addition to displaying a strong sugar-intolerant phenotype upon whole-body knockdown, the fat body-specific loss of *tailless* resulted in developmental delay and reduced survival on HSD (Figs. 4a, 7a and Supplementary Fig. S4). Very little attention has been paid to the function of *tailless* in metabolism. We, therefore, wanted to examine the sugar-intolerant phenotype and function of *tailless* in the fat body in more detail.

We first tested whether *tailless*-knockdown animals exhibited perturbed energy homeostasis and measured the levels of circulating carbohydrates in *tailless*-fat body-specific-knockdown larvae. However, we found that levels of glucose and trehalose were unchanged as compared to controls (Fig. 7b, c). There was also no change in the levels of triglycerides, the most common storage form of lipids in *Drosophila* (Fig. 7d). This indicates that *tailless* controls sugar metabolism via mechanisms other than nutrient storage.

Mice with whole-body knockout of *NR2E1*, the mammalian homologue of *tailless*, have been reported to have reduced body size and adiposity^34^. Interestingly, we found that *tailless*-knockdown did not affect size on HPD, but did stunt growth on HSD (Fig. 7e). This suggests a conserved function for *tailless* in regulating organismal growth, which acts via interaction with specific nutrients. We tested whether the reduced survival, developmental delay, and reduction in size seen in *tailless*-knockdown animals on HSD was due to an aversion towards the high-sugar diet. However, all larvae consumed equal amounts of food regardless of genotype or diet (Fig. 7f), indicating that the sugar-dependent phenotype is indeed due to an inability to tolerate ingested sugar.

### Tailless regulates sugar-responsive expression of *CCHa2*

We next studied the mechanisms by which fat body-specific *tailless* regulates survival and growth. A promising candidate was insulin signalling, a well-conserved pathway governing growth and metabolism. Flies secrete insulin-like peptides (dILPs) from specialized secretory neurons, the insulin-producing cells (IPCs). Secretion of dILPs regulates peripheral insulin signalling pathway activity, with dILP2 being one of the main dILPs regulating larval growth^35^. Impairment in secretion of dILP2 leads to its accumulation in IPCs^36,37^. Indeed, we found that knockdown of *tailless* in the fat body led to a doubling of dILP2 in the IPCs (Fig. 7g), indicating that fat body-specific expression of *tailless* is necessary for dILP2 secretion.

We further examined the fat body-brain communication that could be involved in this remote regulation. A peripheral tissue-derived hormone *CCHamide-2* (*CCHa2*) has been recently shown to respond to nutrients and to control dILP2 secretion in IPCs^38^. We analyzed the expression of *CCHa2* in the fat body and observed elevated expression in response to sugar. Fat-body-specific knockdown of *tailless* prominently inhibited this activation, demonstrating a role for *tailless* in sugar-induced activation of *CCHa2* (Fig. 7h). Next, we asked whether *CCHa2* is required for dietary sugar tolerance by assaying the growth of *CCHa2* mutants on HPD and HSD diets. We backcrossed *CCHa2* mutants^38^ into an inbred w1118 stock for 10 generations to minimize the effects of genomic background. *CCHa2* mutants displayed delayed larval development (Fig. 7i) and significantly reduced pupal volume (Fig. 7j) on HSD, phenocopying *tailless* RNAi flies. To conclude, these results show that the orphan nuclear hormone receptor *tailless* in the fat body regulates organismal growth in response to high dietary sugar content by activating the expression of *CCHa2*.

### Sugar tolerance genes in flies are associated with T2D in humans

**Fig. 8:**
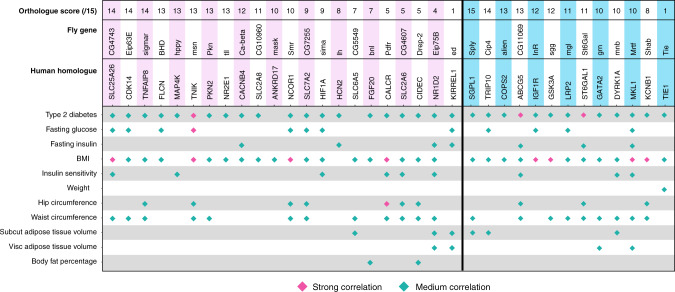
Comparison of hits to human SNP data associated to diabetes phenotypes. The majority of the human homologues of the identified hits are associated with T2D related traits. T2D knowledge portal (https://www.type2diabetesgenetics.org/).

Finally, we examined whether the human homologues of the genes identified are known to be associated with T2D and related traits. We utilized the T2D knowledge portal (http://www.type2diabetesgenetics.org/) that combines data from multiple datasets. Indeed, we found that the human homologues of almost all the genes we identified have been associated with T2D (Fig. 8). We also found the majority of genes to be associated with changes in BMI and waist circumference, and a large number also with fasting glucose. For example, variants of *TNIK*, the closest human homologue of *misshapen*, are associated with T2D, fasting glucose, and BMI. Although many of these genes have been associated with T2D by several human GWAS studies, the mechanisms by which these genes regulate metabolism and/or development of human metabolic disease, have often remained elusive. Our results provide a roadmap to uncovering the mechanistic role of these genes in the development of metabolic diseases.

## Discussion

In this study, we have identified several genetic regulators of macronutrient tolerance in flies. This is significant as other nutrient-responsive genes such as mTOR and PPAR play a central role in disease and have been identified as major therapeutic targets. Here we identify the orphan nuclear hormone receptor *tailless* in the fat body as a regulator of insulin signalling and organismal growth in response to high dietary sugar content. Our finding that *tailless* regulates insulin signalling and growth through dILP2 secretion is compelling given recent findings. Mice with knockout of *NR2E1*, the mammalian homologue of *tailless*, develop insulin resistance and non-alcoholic fatty liver disease, and reduced growth and adiposity, with the phenotype being aggravated on a high-fat diet^39^. Furthermore, knockdown of *NR2E1* in mouse β-cells leads to decreased insulin secretion and proliferation, and increased apoptosis^40^. In humans, *NR2E1* has also been found to correlate with inflammation as well as high-fasting glucose and insulin levels in T2D^41^. This indicates that at least parts of the metabolic functions of *tailless* are conserved in mammals. Intriguingly, *NR2E1* has also been shown in several human cell lines to regulate *Sirt1*^42,43^. *Sirt1* is a well-studied regulator of metabolic homeostasis and has been implicated in the development of insulin resistance and diabetes in mammals and *Drosophila*^44,45^.

We found that *tailless* regulates organismal growth through fat body-specific expression of hormone *CCHa2*, the ligand of receptor *CCHa2R*. Interestingly, loss of *CCHa2R* homologue *Bombesin receptor subtype-3* (*BRS-3*) in mice leads to dysregulation of glucose metabolism and obesity^46^. *BRS-3* is an orphan receptor, and the lack of an endogenous ligand has somewhat hindered further study. However, *BRS-3* has been identified as an important regulator of satiety, metabolic rate and obesity in the brain, glucose uptake in muscles, insulin secretion in β-cells, and glucose uptake and lipogenesis in adipose tissue^46,47^. *BRS-3* action seems to be at least partially conserved in *Drosophila*, as fat body-derived *CCHa2* regulates insulin secretion and growth in flies through IPC-specific *CCHa2R*^38^. Considering our finding that *tailless* regulates *CCHa2* in *Drosophila*, it will be interesting to see whether *NR2E1* contributes to *BRS-3* regulation in mammals.

Among the other genetic regulators of macronutrient tolerance was the dual-specificity glucose and fructose transporter *GLUT8* homologue *CG10960*. *GLUT8*-knockout male mice have been reported to be resistant to HFD- and high-fructose diet-induced obesity and insulin resistance^26^. Moreover, *GLUT8* is transcriptionally induced in the liver in response to fasting, and *GLUT8*-knockout mice show enhanced lipid mobilization, ketogenesis and thermogenesis, and reduced mitochondrial oxidative function during fasting^48^. We found that whole-body knockdown of *CG10960/GLUT8* resulted in lethality in all diets except those containing sucrose (HSD and WD), suggesting that *CG10960/GLUT8* is vital to sugar metabolism in flies. We did not observe any change in the starvation resistance of *CG10960/GLUT8* fat body-, gut- or muscle-RNAi adult flies. However, the *CG10960/GLUT8* whole-body-knockdown larvae showed increased levels of circulating glucose and trehalose, especially on HPD, further showing that these animals have disturbed metabolism in the absence of dietary sucrose.

We also identified two other transporters in our screen, an amino acid transporter *CG7255* and mitochondrial transporter *CG4743*. The closest homologues of *CG7255* are *SLC7A2* and *SLC7A3*, both of which transport the cationic amino acids lysine, arginine, and ornithine. It is intriguing to speculate why an amino acid transporter is required for dietary sugar tolerance, and further studies are required to understand the crosstalk between amino acid transporter(s) and sugar metabolism. *CG4743* is a highly conserved mitochondrial S-adenosylmethionine (SAM) carrier and SAM is the most important methyl donor in eukaryotic cells^49^. Mutations in human SAM carrier *SLC25A26* have been shown to cause mitochondrial defects, including changes in mitochondrial RNA stability, translation, and reduced mitochondrial methylation^50^. The single SAM carrier is believed to be responsible for all SAM entry to mitochondria, and changes in cytoplasmic SAM levels have been proposed to be linked in several human pathologies including aging. How and why dietary sugars depend on the appropriate entry of SAM into the mitochondria remains to be studied.

One of the strongest phenotypes in our functional screen was that of *Protein Kinase N* (*Pkn*). The *Drosophila* genome encodes a single orthologue of *Pkn* that is closely related to the mammalian *PKN2*. *Drosophila* Pkn binds specifically to GTP-activated Rho1 and Rac1 GTPases and is required for the dorsal closure in the developing fly embryo^51^. Recently, both in vitro and in vivo knockdown of *PKN2* in primary human skeletal muscle cells and mouse muscle, respectively, has been shown to decrease glucose uptake^52^. Surprisingly, given the sugar intolerant phenotype of *Pkn* RNAi animals, we did not observe any changes in the HSD-induced transcriptional response of *FAS*, *ACC* or *sugarbabe*. We also observed extremely high metabolic rates in HPD-fed fat body-specific *Pkn*-knockdown adult flies, suggesting that *Pkn* plays a role in energy metabolism in the fat body.

The *Ecdysone inducible protein 75B*, *Eip75B*, encodes a nuclear receptor that is a target of the Ecdysone receptor^53^. *Eip75B* shares homology with the mammalian *PPAR* family of nuclear receptors, and in fact, a known PPAR*γ* activator pioglitazone has been shown to act via Eip75B in *Drosophila*^54^. We found that knockdown of *Eip75B* leads to both dietary sugar intolerance and blunted induction of *FAS* and *ACC* in response to HSD. The induction of *sugarbabe*, which is highly expressed upon HSD by Mondo/Mlx^3^, was however normal in *Eip75B*-knockdown animals suggesting that *Eip75B* regulates HSD-induced de novo lipogenesis at least partially independent of the Mondo/Mlx-sugarbabe axis.

We found that the entire JNK pathway is required for dietary sugar tolerance in flies. *Neural Lazarillo* (*NLaz*), a target of JNK signalling, has been previously shown to be activated by HSD in flies and to regulate circulating glucose levels^55^. The *Drosophila* *TNFα* homologue *eiger* is activated in the fat body upon amino acid restriction. Upon activation, the fat body-derived eiger acts remotely in the brain (IPCs) and activates JNK signalling, leading to the inhibition of dILP secretion^56^. Interestingly, none of the four tested *eiger* RNAi lines resulted in sugar intolerance. This may be due to insufficient knockdown or there might be another, yet unidentified, TNF ligand that activates wengen/grindenwald in response to sugar. JNK signalling has emerged as one of the most studied pathways in regulating obesity and insulin resistance. JNK is activated during obesity and *JNK1* knockout mice are lean and resistant to diet-induced obesity^57^. Our finding that sugar-induced de novo lipogenesis is dependent on *sigmar*, suggests a key role for JNK signalling in fat storage. It has been suggested that some of the pathology of insulin resistance and T2D arises when adipocyte storage capacity is exceeded, and the lipids “overflow” into muscle and liver^58^. The role of JNK signalling in this process remains to be explored.

*Drosophila* has been widely used to study obesity and metabolic disease and flies accumulate triacylglycerol in response to caloric overload similar to mammalian models. Existing fly studies have utilized both high-sugar and high-fat diets to induce obesity. High-sucrose diets have been shown to induce fat accumulation and impaired glucose metabolism in both adult flies and larvae^18,55,59–61^. A lard-based HFD has also been shown to induce lipid accumulation, hyperglycemia, and impaired insulin signalling in adult flies^62^. Coconut oil-based HFD induces lipid accumulation and heart dysfunction in adult flies^63,64^. The use of different diets across studies, although important to study, presents a challenge for comparing results. Moreover, there are substantial discrepancies between laboratories in the so-called “standard diet” used in fly studies. In addition, increasing evidence shows that animal populations and individuals, including fruit flies, differ in their metabolic responses to diet. We have previously identified a number of genes that are crucial for dietary sugar tolerance in flies, and when downregulated, lead to sugar intolerance and impaired development^3,27^. This suggests that there may be a large amount of natural variation in dietary tolerance between fly populations and that this variation should be taken into account in study design.

In this study we used a common base of 10% w/v baker’s yeast across all diets. Sucrose, coconut oil, lard, and starch were then added at around the highest amount believed to be tolerated^27,64^, thus pushing animals to their metabolic limits. We show that even closely related *D. melanogaster* strains display marked variation in their survival under these different diets. Notably, we observed the greatest variation in the survival on high-sucrose and high-coconut oil diets. Coconut oil has been used by a number of groups to examine the effect of high-fat diet in flies^63–68^. In contrast, few studies have used lard as a supplement to create a high-fat diet in fly studies^62,69^. Coconut oil and lard differ greatly in their fatty acid composition. Coconut oil contains about 90% saturated fatty acids, the main one being lauric acid. Coconut oil has only about 9% unsaturated fatty acids and almost all of it is oleic acid. In comparison, lard is composed of ~40% saturated and 59% unsaturated fatty acids. The main saturated fatty acid in lard is palmitic acid and it does not contain any lauric acid. The main unsaturated fatty acid in lard is also oleic acid, but the amount is several times higher (44%) than in coconut oil (7%)^70^. There was very little variation in the survival of DGRP strains on the high-lard diet, and moreover, this diet led to faster development in most of the strains tested. Flies do not have bile nor can they synthesize cholesterol, and the mechanisms by which they utilize dietary fats may differ greatly from mammals. However, our results and other literature suggest that dietary fats play an important role in the development and survival of flies. For example, flies lacking the *LXR* nuclear receptor homologue *Hr96* and its target *lipase A* homologue *magro*, are not able to break down dietary TAGs and are starvation sensitive^71^.

To our surprise, DGRP strains showed great variation in their survival on HSD. In nature fruit flies are attracted to ripened fruits that often have high sugar content. In the laboratory, flies are fed diets ranging from complex mixtures that include molasses, corn meal, malt, soy flour, dextrose, corn syrup, sucrose, glucose, and different yeasts to simple diets composed of just baker’s yeast, sucrose, and agar (also known as SYA diet). As we show here, the source of carbohydrates is important; we found that complex carbohydrates such as starch are more widely tolerated than simple sugars. Our results emphasize that differences in dietary carbohydrate content and source among laboratories could be an important cause of inter-study heterogeneity.

It is widely recognized that diet composition has a dramatic impact on human health. However, there is no clear consensus on what comprises the optimal healthy diet. This is due to the remarkable differences between individuals in their physiological and metabolic responses to nutrients, as well as an incomplete understanding of the complex metabolic pathways and how they interact with different nutrients and diets. Moreover, there is notable variation in dietary behaviour between human populations. Surprisingly, the mechanisms responsible for this inter-individual heterogeneity in responsiveness to different dietary interventions are still poorly understood.

Human GWAS studies have identified thousands of loci contributing to complex human diseases such as obesity and T2D. However, the majority of loci identified show either very modest effects on disease phenotype and/or can be difficult and expensive to verify experimentally in higher model systems. With the exception of monogenic diseases, the development of disease is a result of a complex interplay between various genetic and environmental factors. However, most studies that attempt to map the genetics of complex disease rarely take the environment into consideration. The gene-by-environment interactions may partly explain the inability to detect mutations that account for the majority of T2D in humans because most association studies do not take environmental diversity into account. Despite the observed variation, the majority of mouse studies on T2D have been done using a single inbred strain, C57BL/6, which is known to develop greater obesity and insulin resistance than many other strains^17,72–76^. The predominant use of a single mouse strain in metabolic research has potentially skewed our understanding of the development of T2D and other metabolic diseases.

Personalized nutrition as a preventive health strategy is still in its infancy. Isolating genes that moderate an individual’s response to different nutrients will have an enormous impact on public health, with the potential to facilitate a revolution in the use of food to treat and prevent disease. This study moves us one step closer to this goal and informs further studies on the mechanistic basis for metabolic disease.

## Methods

### Fly food, stocks, and husbandry

196 inbred DGRP lines were obtained from Bloomington Stock Centre. RNAi lines for functional validation screen were obtained from Vienna *Drosophila* Resource Centre and Bloomington Stock Centre. A full list of RNAi lines used is in Supplementary Data File. All stocks were maintained at +25 °C on medium containing agar 1% (w/v), dry baker’s yeast 1.3% (w/v), molasses 8% (w/v), corn flour 4% (w/v), proprionic acid 0.6% (v/v), nipagin 1.2% (v/v).

The experiments took place at +25 °C, 65% humidity under a 12 h:12 h light:dark cycle. The diet screen on 196 DGRP strains was conducted in cohorts of 10 strains, each strain run in 4 replicates of 30 larvae per vial, on all diets at the same time. For defined nutrient studies, larvae were grown on defined food containing 0.5% (w/v) agar, 2.4% (v/v) nipagin, 0.7% (v/v) propionic acid and 10% (w/v) dry baker’s yeast, supplemented with varying concentrations of sucrose (w/v), coconut oil (w/v), lard (w/v) or potato starch (w/v). HPD was composed of 10% (w/v) dry baker’s yeast, and the other diets had the same amount of yeast as a base. HSD was supplemented with 20% (w/v) sucrose, HFDcoco with 20% (w/v) coconut oil, HFDlard with 20% (w/v) lard, WD with 10% (w/v) sucrose and 10% (w/v) lard, and HStD with 20% (w/v) potato starch. First-instar larvae were collected from apple juice plates (apple juice 33.33% (v/v), agar 1.75% (w/v), sucrose 2.5% (w/v) and nipagin (2% (v/v)). Larvae were grown on defined diets at controlled density (30 larvae per vial).

*CCHa2* back crossed mutants were confirmed by T7 endonuclease I assay. Primers used for PCR amplification of the genomic region of interest (~1.2 kb):

CCHa2MuCheck-F: GGCCAAGGGATAATCAAGTTCACC

CCHa2MuCheck-R: GACAACTCCCAAGTGTGCAACACG

Target region of T7 positive lines were then sequenced to validate the CCHa2 mutation.

### BOMB calorimetry

Total energy content of fly diets was obtained by bomb calorimetry calibrated with dry benzoic acid as a thermochemical standard (Parr Instrument Company, Moline, IL USA). Fly diets were pelleted to approximately 0.7 g/pellet, and their wet weight (*w*) recorded. Samples were freeze-dried and the total amount of energy that was lost as heat (∆*t*) measured on a Parr 6100 Oxygen Bomb Calorimeter according to the manufacturer’s instructions. The gross heat of combustion (Hg) of the samples was determined using the calculated energy equivalent (*E*) of the standard in the following formula:

$${{{\rm{Hg}}}}=∆t(E)/w$$

### Metabolic assays

Hemolymph glucose was measured from third-instar pre-wandering larvae using the GAGO-20 kit (Sigma) as described previously^77^. Trehalose was measured after conversion into glucose and relative values were calculated after subtracting free glucose levels.

### Triglycerides

For triglyceride samples pre-wandering third-instar larvae were snap-frozen in liquid nitrogen. The samples were homogenized in cold PBS + 0.5% Tween. Sample protein quantity was determined using Pierce^TM^ BCA protein assay kit (Sigma) according to the manufacturer’s instruction. The rest of the sample was heat-inactivated for 10 min at 70 °C. Triglyceride levels were measured after conversion to free glycerol, after which original free glycerol was subtracted, as described previously^78^.

### Food consumption assay

10 early third-instar larvae raised on HPD or HSD were transferred for 3 h to their respective diets with 0.05% erioglaucine dye (Sigma-Aldrich), weighed, and then snap-frozen in liquid nitrogen. Samples were homogenized in PBS and the absorbance was measured from the supernatant. The amount of food consumption was calculated using a standard curve and normalized to larval weight.

### RNA extraction and qPCR

Five second-instar larvae per sample were homogenized and RNA was extracted using Nucleospin RNA kit (Macherey-Nagel) according to the manufacturer’s protocol. For fat body-specific analysis, third-instar non-wandering larvae were raised on HPD and acutely exposed to HSD (or HPD for control), after which fat bodies from 3 larvae per sample were dissected and RNA was extracted with Nucleospin RNA kit (Macherey-Nagel) according to the manufacturer’s protocol. Reverse transcription was performed with equal amount of RNA (RevertAid H Minus First Strand cDNA Synthesis Kit, Thermo Scientific). qRT-PCR experiment was conducted using Maxima SYBR Green qPCR Master Mix (2X) (Fermentas) in the Light cycler 480 Real-Time PCR System (Roche). See Supplementary Table 5 for primer sequences.

### DAMS starvation

One-day-old male flies were collected and fed HPD or HSD for 3 days (10 males per vial) after which the flies were individually housed in monitor tubes with an outside diameter of 5 mm containing 1% agar in water (PPT5x65, DAM2 *Drosophila* Activity Monitor, Trikinetics, Waltham, MA). 28–32 flies for each diet and genotype were analyzed. The time of death was determined as the last activity bout observed.

### dILP staining

Brains were dissected from non-wandering third-instar larvae raised on standard laboratory diet, fixed in 4% formaldehyde in PBS for 30 min at room temperature, and washed in PBT buffer (0.3 % Triton X 100 in PBS). They were blocked using 5% BSA in PBT buffer for 2 h at room temperature. Samples were incubated with rabbit anti-dILP2 antibodies^79^ at 4 °C overnight. Samples were washed thrice in PBT (15 min each), incubated with goat anti-rabbit antibodies for 2 h at room temperature. Both antibodies were used in the dilution of 1:400. After three washes, they were mounted in Vectashield mounting medium (Vector Laboratories). Images were acquired using the same scan and laser power settings for each IPC cluster with a Leica TCS SP5 MP SMD FLIM confocal laser scanning microscope. Total signal from each cluster was quantified using ImageJ software (NIH).

### Pupal volume

Pupal volume was measured as previously described^80^.

### Tissue-specific pupation kinetics

For tissue-specific developmental analysis, we determined the point at which half of larvae (i.e., 15 of 30) pupated. In order to do this, the data were fitted to a sigmoidal curve using the multipleFitFunction in the sicegar package. The resulting slope, midpoint, and maximum were taken and put into the sigmoidal equation in order to calculate the time it would have taken for 15 to pupate, as:

1 $$I\left(t\right)=\frac{{I}_{{\max }}}{1+{{\exp }}\left(-{a}_{1}\left(t-{t}_{{{{{\rm{mid}}}}}}\right)\right)}$$

where *I* is intensity which represents the number of larvae pupated, *I*_max_ is the maximum reached, *a*_*1*_ is the slope, *t* is time and *t*_mid_ is the time at which the midpoint of the curve is reached, and exp is the natural exponent.

For *I*(*t*) = 15, and rearranging the equation:

2 $$t=\frac{{{{{{\rm{ln}}}}}}\left(\frac{{I}_{{\max }}}{15}-1\right)-{a}_{1}\times {t}_{{{{{{\rm{mid}}}}}}}}{-{a}_{1}}$$

where ln is the natural logarithm.

### GxE

Statistical evidence for the presence of genetic variance in the response of pupation to diet was obtained using generalized linear mixed models (GLMMs) with a binomial family (logit-link function). The response was numbers of animals within a replicate vial that pupated relative to those that failed. Two GLMMs were implemented, GLMM1 included diet as a categorical fixed effect and strain as a random effect, while GLMM2 also included a random slope for diet at the level of each strain. The relative fit of the two models was compared via AIC and a *χ*^2^ test for the reduction in deviance, with a significant improvement in model fit for GLMM2 indicating significant (non-zero) variance in the response to diet among strains (i.e., gene-by-environment interaction). Models were implemented using the ‘glmer’ function in the packages *lme4* and *lmerTest*. GLMMs were compared using the *anova* function in base R.

### GWAS

Genotype information for the strains was downloaded from the DGRP2 website, which contained information for 4,438,427 variants across 205 lines. Following selection of lines for which phenotypic information was measured, and variants in which at least five lines existed in either ‘reference’ and ‘alternate’ alleles, we performed statistical testing on 2,523,373 variants across 196 lines. Lines with missing allele information for a given variant were not considered (Supplementary Fig. S3). We performed statistical testing with unnormalized phenotypic response (pupation and eclosion proportion) across six diets, and with normalized phenotypic response (pupation and eclosion proportion subtracted by HPD pupation and eclosion proportion respectively) across the five other diets. Statistical testing included multivariate analysis of variance (MANOVA) testing, with Wolbachia status as a covariate per variant, as well as Wilcoxon Rank Sum Tests per diet. To assess the effect size, we calculated the median difference in phenotype between ‘reference’ and ‘alternate’ allele groups per diet and phenotype. We selected SNPs according to unadjusted MANOVA *p*-value < 10^−5^, Wilcoxon Rank Sum Test *p*-value < 0.01, and absolute difference in median phenotypes at least 0.3 (unnormalised) or 0.2 (normalized). For pupation rate analysis, we extracted the time for which at least half of the pupated flies would pupate and tested for differences using two-sample t-tests, corrected for multiple testing using the Bonferroni method. To assess the overall level of pupation between diets, we performed paired t-tests between each diet pair and corrected for multiple testing using the Bonferroni method.

### Metabolic rate measurements

The metabolic rate of an adult fly was measured using indirect calorimetry and estimated from its rate of CO_2_ production (${\dot{V}}_{{{{{{{\rm{CO}}}}}}}_{2}}$, μL CO_2_ h^−1^) at 25 ± 1 °C. ${\dot{V}}_{{{{{{{\rm{CO}}}}}}}_{2}}$ was measured using an 8-channel, flow-through respirometry system following our previously published protocols^81^. Measurements were conducted over a period of seven days, and flies were 2–6 days of age at the time of measurement. To convert rates of CO_2_ production to rates of energy metabolism we assumed that flies were catabolising the same mixture of substrates that was in their diet, and thus the respiratory quotient (RQ: the ratio of CO_2_ production to O_2_ consumption) of flies would reflect this substrate composition. We used a relationship between RQ and dietary sugar-to-yeast (S:Y) ratio derived from a previously published work^82^ to predict the RQ for the two S:Y ratios used in the present study (RQ = 0.872 + 0.125log_10_(S:Y ratio + 1), *t*_63_ = 7.58, *p* < 0.001). We then estimated the energy equivalent of CO_2_ for our two diets by linearly interpolating between the values for mixed protein catabolism for a uricotelic species (25.4 J mL^−1^, RQ = 0.74) and carbohydrate catabolism (21.1 J mL^−1^, RQ = 1)^83,84^ (energy equivalent of CO_2_ = 37.64 – 16.54RQ). We then used these energy equivalents to convert our measured rates of CO_2_ production to estimates of rates of energy metabolism (mJ h^−1^). Finally, to account for variation in metabolic rate associated with variation in activity and body mass, we fit a linear model to our metabolic rate data that included experimental condition, body mass, and activity as fixed factors. Metabolic rate was positively correlated with body mass (parameter estimate ± SE: 39.35 ± 11.12, *t*_147_ = 3.54, *p* < 0.001) and activity (parameter estimate ± SE: 7.64 ± 0.60, *t*_147_ = 12.72, *p* < 0.001). The parameter estimates for mass and activity from this linear model were used to standardize metabolic rate data to the mean fresh mass of control flies fed a HPD within each tissue-specific library (GD = 0.83 mg, kk = 0.77 mg, Trip = 0.81 mg) and to zero levels of activity. These adjusted measures of metabolic rate can therefore be interpreted as mass-independent values for inactive animals, which we will henceforth refer to as resting metabolic rate.

### Reporting summary

Further information on research design is available in the Nature Research Reporting Summary linked to this article.

## Supplementary information

Supplementary Information

Description of Additional Supplementary Information

Supplementary Data 1

Reporting Summary

## Data Availability

Data generated and analyzed in this study are included in this published article and its supplementary information files. T2D knowledge portal (http://www.type2diabetesgenetics.org/) was used for Fig. 8. In addition, Source data are provided as a Source Data File. All other data are also available from the corresponding author upon requests and at https://github.com/AlistairMcNairSenior/DGRP_Diet_Pupation. An archived release of the repository is available 10.5281/zenodo.5895053^85^. Source data are provided with this paper.

## Source data

Source Data

## Code availability

All R code is available at https://github.com/AlistairMcNairSenior/DGRP_Diet_Pupation. An archived release of the repository is available 10.5281/zenodo.5895053^85^.
