# Supplementary material for: Genetic variation of macronutrient tolerance in Drosophila melanogaster
Source: Nat Commun. 2022 Mar 28;13:1637. doi: 10.1038/s41467-022-29183-x (PMC8960806; doi:10.1038/s41467-022-29183-x)
Supplement: Supplementary file 1 — Supplementary Information [file 41467_2022_29183_MOESM1_ESM.pdf]

Supplementary information

Genetic variation of macronutrient tolerance in *Drosophila melanogaster*

Havula E., Ghazanfar S., Lamichane N., Francis D., Hasygar K., Liu Y., Alton L.A., Johnstone J., Needham E.J., Pulpitel T., Clark, T., Niranjan H.N., Shang V., Tong, V., Jiwnani N., Audia G., Alves A.N., Sylow L., Mirth C., Neely G.G., Yang J., Hietakangas V., Simpson S.J. and Senior A.M.

This file contains:

- Supplementary Figure 1
- Supplementary Figure 2
- Supplementary Figure 3
- Supplementary Figure 4
- Supplementary Figure 5
- Supplementary Table 1
- Supplementary Table 2
- Supplementary Table 3
- Supplementary Table 4
- Supplementary Table 5

Supplementary Figure 1

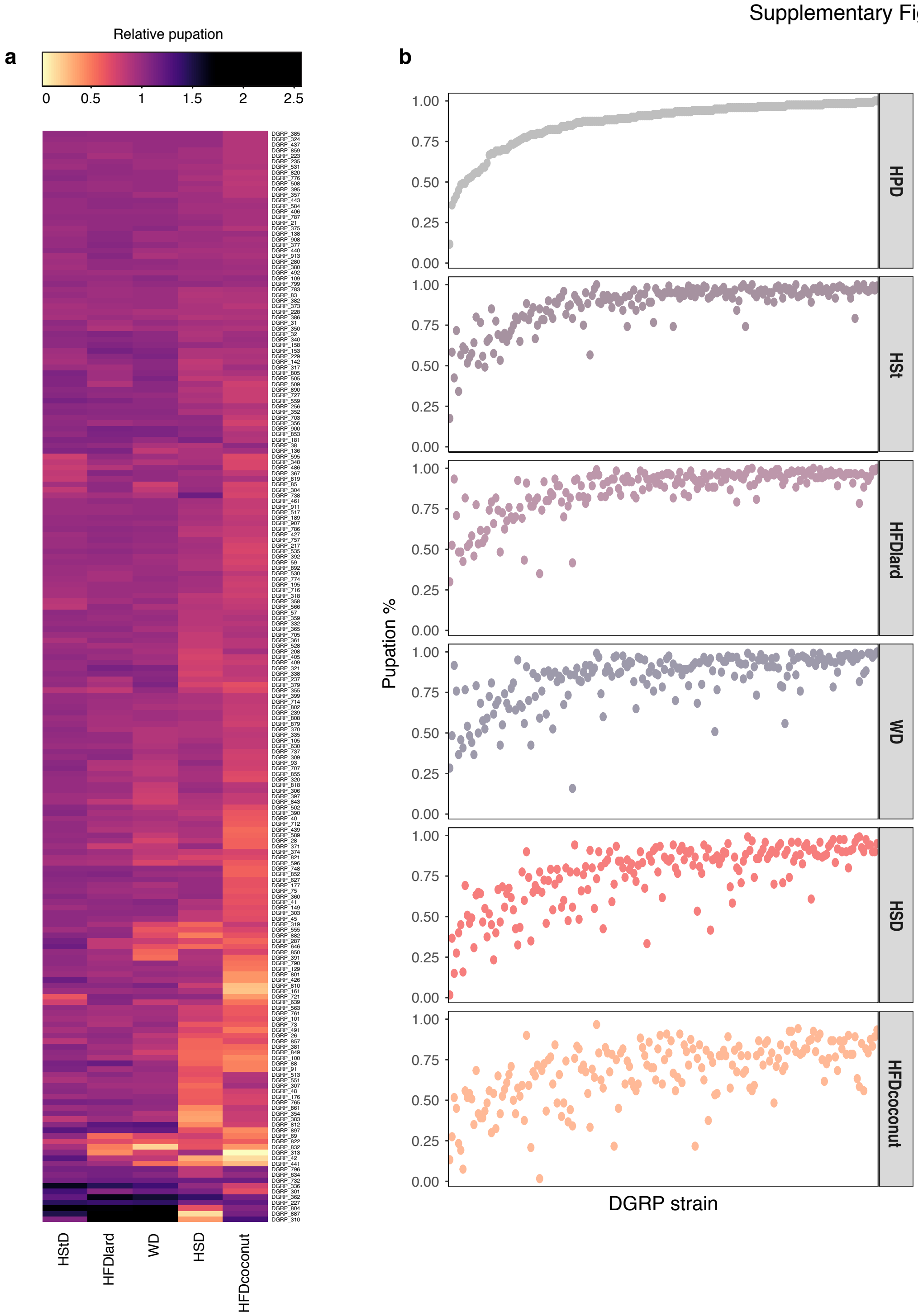

Supplementary Figure 1

(a) A heatmap showing the relative survival (normalized to HPD) of the 196 DGRP strains into pupal stage. **(b)** Pupation % (ordered) of 196 DGRP strains. Source data are provided as a Source Data File.

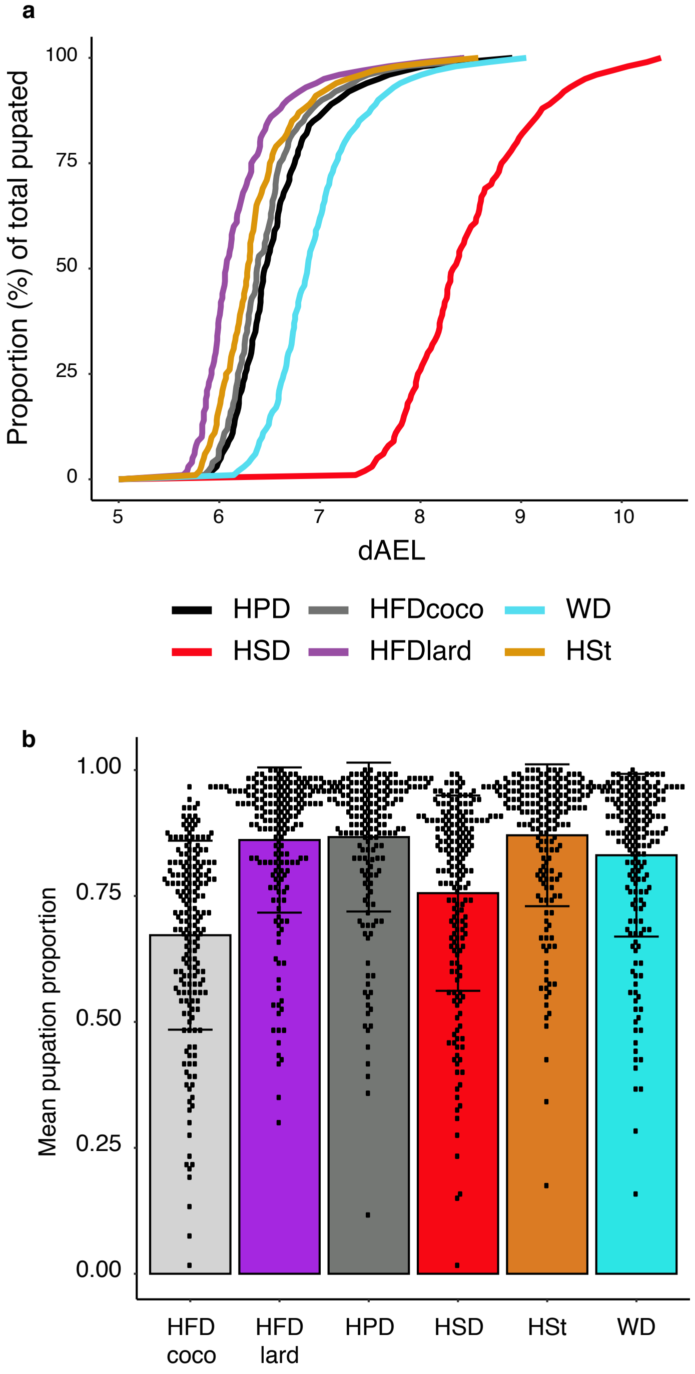

Supplementary Figure 2

(a) Proportion (%) of pupation across time (days) of 196 DGRP strains per diet. Overall survival comparison compared to HFDcoco, HSD, and WD paired two-sided t-test Bonferroni adjusted p-value < 0.05, no significant difference compared to HPD or HStD. Pupation kinetics test of half-life days (time at which 50% of pupated flies have achieved pupation) showed significantly faster pupation on HFDlard compared to all other diets, two-sample t-test Bonferroni adjusted p < 0.05. **(b)** Mean pupation proportion (%) of each 196 DGRP strains per diet. dAEL = days after egg laying

Supplementary Figure 3

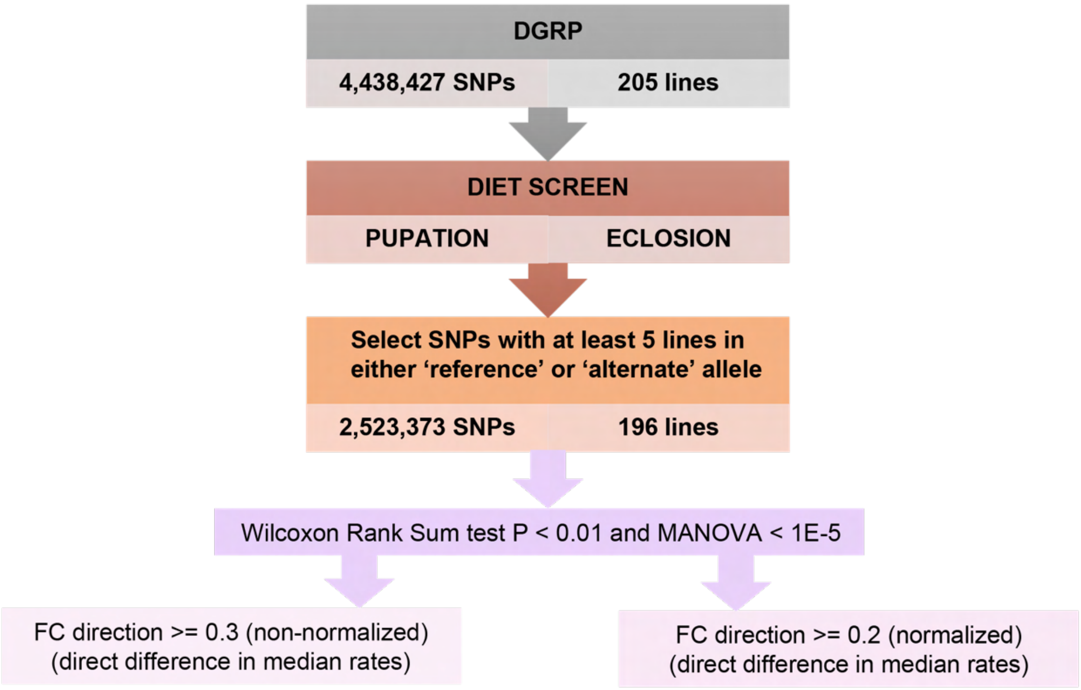

FC direction >= 0.3 (non-normalized)

PUPATION

	Higher in reference (negative) / Unique to diet	Higher in alternate positive) / Unique to diet
**HPD**	72 / 6	0 / 0
**HSD**	173 / 69	3 / 1
**HFDcoco**	157 / 62	1 / 1
**HFDlard**	167 / 40	5 / 3
**WD**	163 / 36	4 / 2
**HStD**	64 / 15	2 / 0

ECLOSION

	Higher in reference (negative) / Unique to diet	Higher in alternate positive) / Unique to diet
**HPD**	145 / 138	1 / 1
**HSD**	96 / 91	3 / 3
**HFDcoco**	20 / 19	0 / 0
**HFDlard**	1 / 1	0 / 0
**WD**	0 / 0	0 / 0
**HStD**	8 / 3	0 / 0

FC direction >= 0.2 (normalized)

PUPATION

	Higher in reference (negative) / Unique to diet	Higher in alternate positive) / Unique to diet
**HPD**	NA	NA
**HSD**	53 / 49	149 / 145
**HFDcoco**	109 / 105	3 / 2
**HFDlard**	6 / 4	2 / 1
**WD**	34 / 29	4 /4
**HStD**	0 / 0	0 / 0

ECLOSION

	Higher in reference (negative) / Unique to diet	Higher in alternate positive) / Unique to diet
**HPD**	NA	NA
**HSD**	19 / 17	13 / 8
**HFDcoco**	20 / 18	244 / 212
**HFDlard**	1 / 0	87 / 52
**WD**	1 / 1	23 / 8
**HStD**	2 / 2	28 / 11

Supplementary Figure 3

GWAS workflow. Source data are provided as a Source Data File.

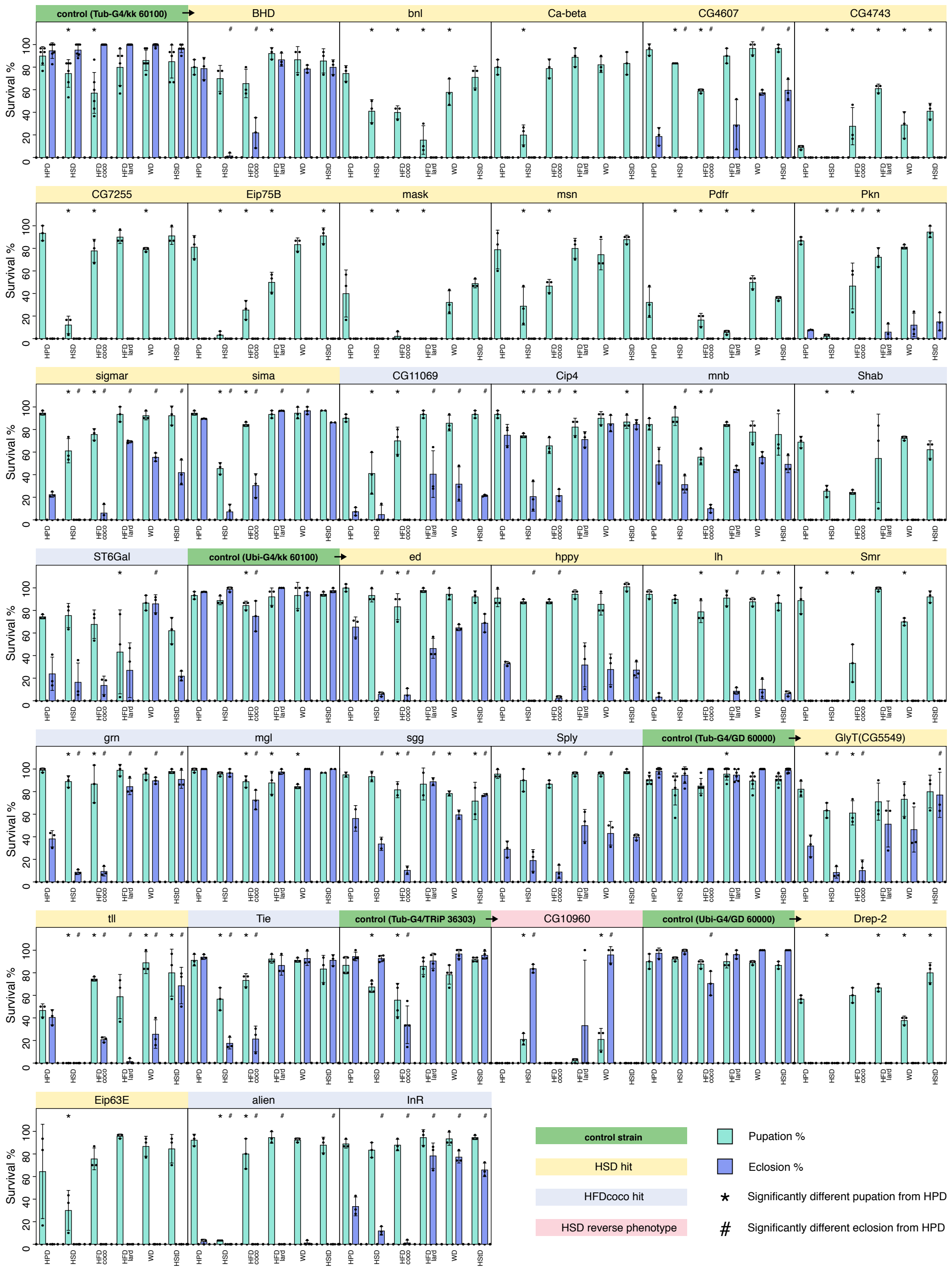

Supplementary Figure 4

The survival (pupation and eclosion) of the Hits identified in the *in vivo* screen across all six diets. *n* = minimum of 3 vials (each with 30 larvae) per diet and genotype. The control is always presented first followed by RNAi lines used on the respective control background. Data are presented as mean values +/- SD. * = Significantly different pupation from HPD, # = Significantly different eclosion on HPD. Significance ($p < 0.05$) is based on coefficients from beta-regression with HPD as the reference diet (equivalent to a two-sided test, unadjusted). See Source Data file for statistical analyses.

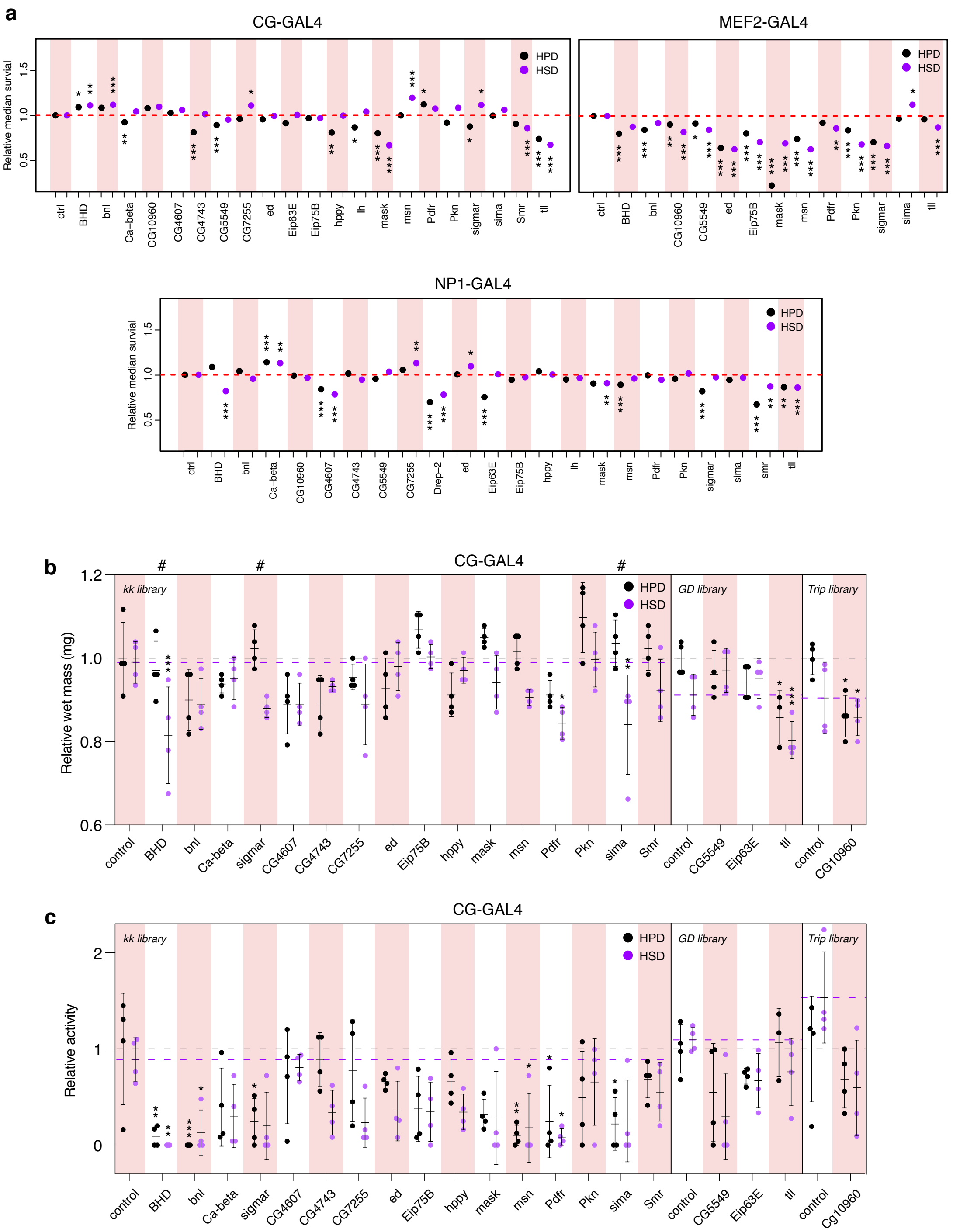

Supplementary Figure 5

(a) Relative survival of adult male flies under starvation upon tissue specific knockdowns (*Cg-GAL4*, *Mef2-GAL4* and *NP1-GAL4*) of HSD hits. *n* = minimum of 28 flies per diet and genotype. See Source Data file for statistical analyses. **(b)** Relative wet mass and **(c)** activity of *Cg-GAL4* knockdown of

HSD hits. $n = 4$ flies per diet and genotype. Statistical significances for (a) were calculated using the Cox proportional hazards regression model (survival package, R; equivalent to a two-sided test, unadjusted), and for (b) and (c) using the two-way ANOVA in conjunction with Dunnett's multiple comparisons test (by genotype) and with Šídák's multiple comparisons test (by diet). See Source Data file for exact *p*-values. Data are presented as mean values +/- SD. ∗ $p < 0.05$, ∗∗ $p < 0.01$, *** $p < 0.001$ (Dunnett's multiple comparisons test), # $p < 0.05$ (Šídák's multiple comparisons test). Source data are provided as a Source Data File.

Supplementary Table 1.

BOMB Calorimetry data of the six experimental diets, and two commonly used "standard laboratory diets"; molasses and dextrose diets.

Sample	wet weight (g)	dry weight (g)	Δt (°C)	Total Hg (MJ/kg)	mean Hg (Mj/Kg)
**HPD**	0.736	0.0798	0.1643	2.2247947	**2.25384067**
**HSD**	0.718	0.2054	0.3442	4.7776751	**4.90509232**
**HFDcoco**	0.7094	0.2113	0.6446	9.05585184	**9.25095745**
**HFDlard**	0.783	0.2275	0.7272	9.2559777	**9.39699798**
**WD**	0.6832	0.1827	0.3904	5.69497921	**6.09141948**
**HStD**	0.7569	0.1169	0.2982	3.92644325	**3.98342904**
**Molasses**	0.7806	0.1184	0.1968	2.51261957	**2.56061502**
**Dextrose**	0.6532	0.1404	0.2441	3.72436121	**3.68510402**

Supplementary Table 2.

Fixed and random effect estimates from GLMM1, which estimates homogeneous responses of pupation to diet among different DGRP strains.

Fixed Effects				
Coefficient	Estimate	SE	z	p
$Intercept_{HPD}$	2.18	0.07	31.24	2.94×10^{-214}
HFDcoco – HPD	-1.35	0.03	-52.71	$<2.23 \times 10^{-308}$
HSD – HPD	-0.86	0.03	-32.80	5.25×10^{-236}
HFDlard – HPD	-0.06	0.03	-1.94	0.05
HStD – HPD	0.03	0.03	1.12	0.24
WD – HPD	-0.33	0.03	-11.72	1.00×10^{-31}
Random Effects (Strain/Genetic Variance)				
Coefficient	Variance	SD		
Intercept	0.86	0.93		

Supplementary Table 3.

Fixed and random effect estimates from GLMM2, which estimates among-strain variance in the response of pupation to diet.

Fixed Effects				
Coefficient	Estimate	SE	z	p
$Intercept_{HPD}$	2.35	0.09	26.06	9.67×10^{-150}
HFDcoco – HPD	-1.53	0.07	-21.02	4.41×10^{-98}
HSD – HPD	-0.96	0.06	-16.34	5.30×10^{-60}
HFDlard – HPD	-0.09	0.06	-1.52	0.13
HStD – HPD	0.03	0.05	0.59	0.56
WD – HPD	-0.36	0.07	-4.84	1.27×10^{-6}
Random Effects (Strain/Genetic Variance)				
Coefficient	Variance	SD	$Corr_{Intercept}$	
$Intercept_{HPD}$	1.45	1.20	NA	
HFDcoco – HPD	0.84	0.92	-0.65	
HSD – HPD	0.46	0.68	-0.37	
HFDlard – HPD	0.47	0.69	-0.36	
HStD – HPD	0.27	0.52	-0.24	
WD – HPD	0.79	0.89	-0.39	

Supplementary Table 4.

Relative fit of GLMM1 and GLMM2. *k* = number of parameters, AIC = Akaike Information Criterion, logLik = log likelihood, DF = degrees of freedom in χ^2 distribution used for test of improvement in model fit for GLMM2.

Model	*k*	AIC	logLik	Deviance	χ^2	DF	p
GLMM1	7	24863	-12425	24849			
GLMM2	27	21452	-10699	21398	3451	20	$<2.23 \times 10^{-308}$

Supplementary Table 5.

qPCR primers used in the study.

Act F:	CCGTACCACAGGTATCGTGTTG
Act R:	GTCGGTTAAATCGCGACCG
CDK7 F:	GGGTCAGTTTGCCACAGTTT
CDK7 R:	GATCACCTCCAGATCCGTG
RP49 F:	AGGGTATCGACAACAGAGTG
RP49 R:	CACCAGGAACTTCTTGAATC
Eip75B F:	ATCTGCATGTTTGACTCGTCG
Eip75B R:	TCCGCGAAATTGAAGGTGGAG
sigmar F:	AGTGGCGCTGAAGTCGATTG
sigmar R:	GCCTCCTCGCCGAAGAAATC
ACC F:	GGCTATGCTGCGCTTAACA
ACC R:	GCCTCTGTTTTGTGGGTGAC
FASN1 F:	CTCCACCATCGAGGAGTTCA
FASN1 R:	CTTGAGCTTGCCAATCCTGT
Pkn F:	GCCATAGCCGTGATGCGTAG
Pkn R:	ATGCCTGTTTCTTAACATCCTC
sugarbabe F:	CCAGCGATTTCGTATGCAACT
sugarbabe R:	GCGGCAATAGTAGAGTCCGTC
CCHa2 F:	GCCTACGGTCATGTGTGCTAC
CCHa2 R:	ATCATGGGCAGTAGGCCATT
