# Supplementary material for: Genetic variation of macronutrient tolerance in Drosophila melanogaster
Source: Nat Commun. 2022 Mar 28;13:1637. doi: 10.1038/s41467-022-29183-x (PMC8960806; doi:10.1038/s41467-022-29183-x)
Supplement: Supplementary file 2 — Description of Additional Supplementary Information [file 41467_2022_29183_MOESM2_ESM.pdf]

Description of Additional Supplementary Information

File name: Supplementary Data 1.

Description: Results of the functional validation screen.
