# Supplementary material for: Genetic variation of macronutrient tolerance in Drosophila melanogaster
Source: Nat Commun. 2022 Mar 28;13:1637. doi: 10.1038/s41467-022-29183-x (PMC8960806; doi:10.1038/s41467-022-29183-x)
Supplement: Supplementary file 4 — Reporting Summary [file 41467_2022_29183_MOESM4_ESM.pdf]

nature portfolio

Corresponding author(s): Essi Havula, Alistair Senior

Last updated by author(s): Feb 25, 2022

Reporting Summary

Nature Portfolio wishes to improve the reproducibility of the work that we publish. This form provides structure for consistency and transparency in reporting. For further information on Nature Portfolio policies, see our Editorial Policies and the Editorial Policy Checklist.

Statistics

For all statistical analyses, confirm that the following items are present in the figure legend, table legend, main text, or Methods section.

n/a	Confirmed	
☐	☒	The exact sample size (*n*) for each experimental group/condition, given as a discrete number and unit of measurement
☐	☒	A statement on whether measurements were taken from distinct samples or whether the same sample was measured repeatedly
☐	☒	The statistical test(s) used AND whether they are one- or two-sided<br>*Only common tests should be described solely by name; describe more complex techniques in the Methods section.*
☐	☒	A description of all covariates tested
☐	☒	A description of any assumptions or corrections, such as tests of normality and adjustment for multiple comparisons
☐	☒	A full description of the statistical parameters including central tendency (e.g. means) or other basic estimates (e.g. regression coefficient) AND variation (e.g. standard deviation) or associated estimates of uncertainty (e.g. confidence intervals)
☐	☒	For null hypothesis testing, the test statistic (e.g. *F*, *t*, *r*) with confidence intervals, effect sizes, degrees of freedom and *P* value noted<br>*Give P values as exact values whenever suitable.*
☒	☐	For Bayesian analysis, information on the choice of priors and Markov chain Monte Carlo settings
☐	☒	For hierarchical and complex designs, identification of the appropriate level for tests and full reporting of outcomes
☒	☐	Estimates of effect sizes (e.g. Cohen's *d*, Pearson's *r*), indicating how they were calculated

Our web collection on statistics for biologists contains articles on many of the points above.

Software and code

Policy information about availability of computer code

Data collection	Starvation data was collected using the commercially available DAM system. This has been fully described in text.
Data analysis	GraphPad Prism 9, Image J and the open source software R version 4.1.0. were used to analyze data. This has been fully described in text and code made available at https://github.com/AlistairMcNairSenior/DGRP_Diet_Pupation.

For manuscripts utilizing custom algorithms or software that are central to the research but not yet described in published literature, software must be made available to editors and reviewers. We strongly encourage code deposition in a community repository (e.g. GitHub). See the Nature Portfolio guidelines for submitting code & software for further information.

Data

Policy information about availability of data

All manuscripts must include a data availability statement. This statement should provide the following information, where applicable:

- Accession codes, unique identifiers, or web links for publicly available datasets
- A description of any restrictions on data availability
- For clinical datasets or third party data, please ensure that the statement adheres to our policy

Data generated and analyzed in this study are included in this published article and its supplementary information files. In addition, Source data are provided with this paper. T2D knowledge portal (http://www.type2diabetesgenetics.org/) was used for Figure 8. All other data are also available from the corresponding author and at https://github.com/AlistairMcNairSenior/DGRP_Diet_Pupation. An archived release of the repository is available DOI: 10.5281/zenodo.5895053.

Field-specific reporting

Please select the one below that is the best fit for your research. If you are not sure, read the appropriate sections before making your selection.

☒ Life sciences ☐ Behavioural & social sciences ☐ Ecological, evolutionary & environmental sciences

For a reference copy of the document with all sections, see nature.com/documents/nr-reporting-summary-flat.pdf

Life sciences study design

All studies must disclose on these points even when the disclosure is negative.

Sample size	Sample sizes were not determined before the experiments. Sample sizes were chosen according to the standards in the field, based on our previously published work (Havula et al. 2013 PLoS Genetics , Mattila et al. 2015 Cell Reports) and on the consistency of measurable differences between groups.
Data exclusions	No data was excluded.
Replication	Replication was extensive throughout the study and key experiments were repeated at least wice. All replications were successful unless otherwise stated in the manuscript.
Randomization	Drosophila lines were allocated to the original screen based on their line number. The screen was run in cohorts of 10 strains, each tested across all diets at the same time. In the case of a general viability issue of a stock, the screen was not halted, but instead the next healthy strain was included into the cohort. Strains with general viability issues were not tested until deemed healthy (no mold growth in vials etc.). More details have been included in the point-by-point response (including analysis of batch effect).
Blinding	Investigators were not blinded during data collection (blinding is not typically used in this type of data collections, which are not subject to experimenter bias). Instead, the biological groups were well defined and we relied upon replication.

Reporting for specific materials, systems and methods

We require information from authors about some types of materials, experimental systems and methods used in many studies. Here, indicate whether each material, system or method listed is relevant to your study. If you are not sure if a list item applies to your research, read the appropriate section before selecting a response.

Materials & experimental systems

n/a	Involved in the study	
☐	☒	Antibodies
☒	☐	Eukaryotic cell lines
☒	☐	Palaeontology and archaeology
☐	☒	Animals and other organisms
☒	☐	Human research participants
☒	☐	Clinical data
☒	☐	Dual use research of concern

Methods

n/a	Involved in the study	
☒	☐	ChIP-seq
☒	☐	Flow cytometry
☒	☐	MRI-based neuroimaging

Antibodies

Antibodies used	1. Rabbit anti-dILP2 antibody was a generous gift from Ernst Hafen, ETH Zürich, Switzerland.<br>2. Goat anti-Rabbit IgG (H+L) Cross-Adsorbed Secondary Antibody, Alexa Fluor 633. Supplier: Invitrogen. Catalog number: A21070. Clone name: N/A. Lot number: 1120101
Validation	1. Rabbit anti-dILP1 antibody has been validated for the use of immunofluorescent analysis of Drosophila insulin producing cells. Bader, R. et al. The IGFBP7 homolog Imp-L2 promotes insulin signaling in distinct neurons of the Drosophila brain. J Cell Sci 126, 2571-2576, doi:10.1242/jcs.120261 (2013).<br>2. Goat anti-Rabbit IgG: https://www.thermofisher.com/antibody/product/Goat-anti-Rabbit-IgG-H-L-Highly-Cross-Adsorbed-Secondary-Antibody-Polyclonal/A-21071

Animals and other organisms

Policy information about studies involving animals; ARRIVE guidelines recommended for reporting animal research

Laboratory animals	For all experiments, mixed-sex groups of 0-20 day old Drosophila Drosophila melanogaster flies were used. The strains used in this study, included the DGRP panel and VDRC and BDSC stocks (all specified in the manuscript).

Wild animals	No wild animals were used in the study.
Field-collected samples	No field collected samples were used in the study.
Ethics oversight	Ethics approval was not required for experiments on invertebrates.

Note that full information on the approval of the study protocol must also be provided in the manuscript.
